# Unveiling the potential of biomechanics in pioneering innovative strategies for cancer therapy

**DOI:** 10.7150/thno.108605

**Published:** 2025-02-10

**Authors:** Xiaodong Wu, Weidong Fei, Tao Shen, Lei Ye, Chaoqun Li, Siran Chu, Mingqi Liu, Xiaodong Cheng, Jiale Qin

**Affiliations:** 1 Women's Hospital, Zhejiang University School of Medicine, Hangzhou, 310006, China.; 2 Zhejiang University School of Medicine, Hangzhou, 310000, China.; 3 Zhejiang Key Laboratory of Precision Diagnosis and Therapy for Major Gynecological Diseases, Hangzhou, 310006, China.; 4 Zhejiang Provincial Clinical Research Center for Gynecological Diseases, Hangzhou, 310006, China.

**Keywords:** biomechanics, cancer, mechanosensors, mechanosignaling proteins, nanotherapeutics

## Abstract

Mechanical force transmission is pivotal in tumor biology, profoundly affecting cancer cell behaviors such as proliferation, metastasis, and resistance to therapy. To explore novel biomechanical-based therapeutic strategies for cancer treatment, this paper deciphers the advances in biomechanical measurement approaches and the impact of biomechanical signals on fundamental oncological processes such as tumor microenvironment remodeling, angiogenesis, metastasis, and drug resistance. Then, the mechanisms of biomechanical signal transduction of tumor cells are demonstrated to identify novel targets for tumor therapy. Additionally, this study proposes a novel tumor treatment strategy, the biomechanical regulation tumor nanotherapeutics, including smart biomaterials designed to disturb mechanical signaling pathways and innovative nanodrugs that interfere transduction of biomechanical signals to improve tumor therapeutic outcomes. These methods mark a departure from conventional pharmacological therapies to novel strategies that utilize mechanical forces to impede tumor progression and enhance tumor responsiveness to treatment. In general, this review highlights the critical role of biomechanical signals in cancer biology from a holistic perspective and underscores the potential of biomechanical interventions as a transformative class of therapeutics. By integrating mechanobiology into the development of cancer treatments, this paper paves the way for more precise and effective strategies that leverage the inherent physical properties of the tumor microenvironment.

## Introduction

Despite significant advancements in anti-cancer drug development, diagnostic methods, and treatment approaches, cancer continues to be the leading cause of mortality worldwide. Cancer has traditionally been understood as primarily stemming from genetic abnormalities, which trigger epigenetic changes and result in abnormal cellular behaviors 1. However, the role of cellular physical properties in cancer initiation and progression has recently attracted scientific attention. Biomechanics, especially the mechanical microenvironment of tumors, plays an important role in cancer prediction, diagnosis, and treatment. Tumor cells inhabit abnormal mechanical microenvironments, including altered solid tumor stress, extracellular matrix (ECM) stiffness, and hydrostatic pressure 2. Tumor cells sense and convert these mechanical signals into biochemical signals through mechanosensors, which include glycocalyx, primary cilia, cytoskeleton, and nucleus. Any disturbance to this mechanotransduction may result in tumor progression. Many biophysicists can predict cellular activities such as division, proliferation, metastasis, and drug resistance through the physical characteristics of tumor cells accurately 3, 4. Therefore, integrating theoretical and experimental approaches from mechanics and biology into tumor biomechanics allows for a detailed investigation of the complex mechanical dynamics underlying cancer 5.

To achieve the transition from specific mechanosensitive (MS) molecules to tumor mechanomedicine, this article elucidates how the tumor mechanical environment impacts their growth and progression, from molecular and subcellular to cellular, tissue, organ, and even whole-body scales perspective. Firstly, this paper elucidates the biomechanical measurement approaches, biomechanical characteristics of tumor tissue, and how biomechanics promote tumor progression. Then, the manuscript decodes the biomechanical signaling mechanisms of cancer cells. The cellular mechanosensors in tumor cells, like glycocalyx and primary cilia, are responsible for sensing mechanical signals in the tumor microenvironment. After that, diverse mechanosignaling proteins are responsible for transmitting biomechanical signals inside tumor cells to achieve better survival. During the above process, this article summarizes a series of potential or clinically studied anti-tumor small molecule drugs that interfere with mechanical signal reception or conduction (**Table 1**). Based on the published research, this paper summarizes a novel tumor treatment strategy: the biomechanical regulation tumor nanotherapeutics, which aims to achieve tumor treatment by blocking the biomechanical signal transduction through the nanosystems. By adopting a holistic, interdisciplinary, rigorous investigation into the biomechanics of cancer, there exists a substantial opportunity to transform established therapeutic strategies. This could significantly boost cancer treatment efficacy, ultimately elevating survival rates and enriching the life quality of patients.

## Biomechanical measurement approaches

Accurate measurement of mechanical properties is crucial for studying tumor mechanobiology. Based on the spatial and temporal scales, as well as the force sensitivity characteristics of tumor tissue, various biophysical techniques, such as atomic force microscopy (AFM), micropipette aspiration (MPA), and traction force microscopy (TFM), have been developed to measure stiffness, viscoelasticity, or deformability, shedding light on the mechanics of tumor cells 5. To fully understand the biomechanical landscape of cancer, it is important to integrate multiple techniques, each tailored to specific research needs. AFM is a widely used method due to its high spatial resolution and ability to measure forces at the nanoscale. It can assess mechanical properties such as Young's modulus, viscosity, surface tension, and adhesion forces in both normal and pathological tissues 6, 7. However, AFM is low-throughput, requires technical expertise, and has limitations related to scan quality and time. MPA involves pulling a cell into a micropipette using negative pressure, with the resulting deformation measured to determine properties such as Young's modulus, surface tension, and intracellular pressure 5, 8. While inexpensive, MPA has low spatial and temporal resolution, and the quality of the seal between the cell and the pipette can significantly affect the results 9. Optical tweezers use focused laser beams to manipulate small objects and apply forces in the femtonewton to piconewton range. This technique is ideal for molecular force analysis, as it allows for precise control of low forces. It is useful for studying mechanical compliance, adhesion forces, and surface tension at the molecular level 5. However, it is inherently low-throughput, as each object is manipulated individually 10. TFM measures the forces of cells exert on an elastic surface. By analyzing the deformation of the substrate, the forces exerted by the cells can be quantified. This technique does not require chemical perturbations, allowing for natural quantification of cellular stresses. However, it requires accurate imaging of cell-substrate interactions and computational methods to analyze the data 5.

Although these methods yield valuable insights into mechanical properties, they are constrained by limitations such as spatial resolution, throughput, and the requirement for direct physical contact with the sample. To overcome these issues, non-contact optical techniques, such as brillouin microscopy, have emerged. Additionally, ultrasound 11 and magnetic resonance imaging 12 have been used to collect mechanical data non-invasively. While these methods are non-invasive, they have lower spatial resolution compared to other techniques, making them less effective for cellular and subcellular analysis. Furthermore, mechanical properties differ across cell types 13. For instance, varying collagen/GAG compositions in different cartilage types result in distinct mechanical characteristics 14. In summary, while current techniques provide valuable information about the mechanical properties of cells and tissues, each has its strengths and limitations.

## The role of biomechanics in tumor progression

### Biomechanical modulation of the tumor microenvironment

**Figure 1:**
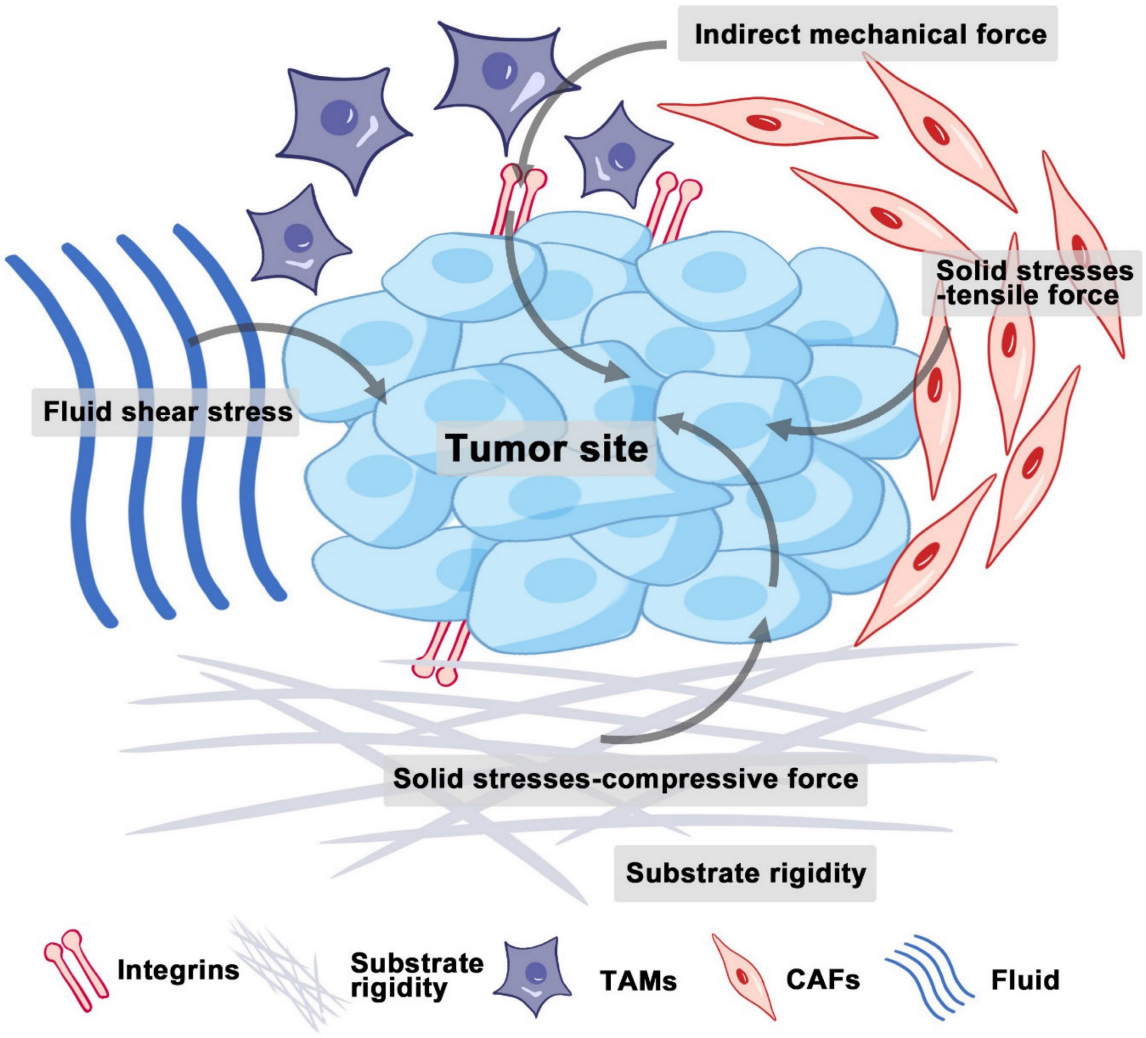
Mechanical forces at the tumor site. Solid stresses encompass both tensile and compressive forces. Increased fluid and hydrostatic pressure result from fluid extravasation from blood vessels and secretions from stromal cells. Indirect mechanical forces are relayed by CAFs and TAMs to mechanosensors. Abbreviations: CAFs, cancer-associated fibroblasts; TAMs, tumor-associated macrophages. (Adapted with permission from Ref. 163. Copyright 2020 Ivyspring International Publisher)

The tumor microenvironment (TME) composed of interstitial cells and ECM is characterized by a complex interaction between cells. The ECM is primarily composed of intercellular matrix and basement membrane, while the interstitial cells include fibroblasts and immune cells (**Figure 1**). The intercellular matrix, which includes components such as collagen, fibronectins, integrins, laminins, and matrix metalloproteinases (MMPs), plays a crucial role in mediating mechanical properties and is a key element of the mechanical signaling pathway 15. Unlike normal tissues, tumor tissues have a unique microenvironment characterized by abnormal structures of blood and lymphatic vessels, increased stromal pressure, and a dense interstitial matrix. Recent studies indicate that, in addition to biochemical cues, physical signals from the microenvironment can play a crucial role in influencing cellular behaviors, including growth, metastatic potential, and drug resistance 2. These physical signals mainly include solid stresses, fluid shear stresses, and indirect mechanical forces (**Figure 1**).

Solid stress accumulates in tumors as proliferating cancer cells exert strain on the surrounding structural elements of both tumor and normal tissues 16. Solid stresses are produced by mechanisms such as cell infiltration, proliferation, matrix deposition, osmotic swelling of glycosaminoglycans like hyaluronic acid (HA), and actomyosin-mediated cell contractions 17. A portion of this stress arises from reciprocal forces imposed by adjacent normal tissue, while the remainder is stored within the cells and matrix components of the tumor. This residual stress, also known as growth-induced solid stress, persists even after tumor excision and the removal of external forces 16, 18. Elevated solid stresses within tumors compress blood vessels, reducing blood flow. Concurrently, the excessive deposition and cross-linking of ECM components, such as collagen, lead to ECM remodeling and thus increased stiffness 19. Therefore, tumors are always appreciably stiffer than normal tissue 20.

Interstitial fluid, composed of water and solutes such as soluble carbohydrates and plasma proteins, exists alongside a solid phase formed by the extracellular matrix. The hydrostatic pressure of this interstitial fluid is referred to as interstitial fluid pressure (IFP) or interstitial hydraulic pressure. Fluid shear stresses encompass microvascular and IFP alongside shear forces exerted by blood and lymphatic flow on vessel walls and by interstitial flow on cancer and stromal cells 21. Elevated IFP, a distinctive feature of solid tumors, results from both solid stress and fluid buildup in the interstitial space 22, potentially influencing tumor cell migration through autocrine C-C chemokine receptor 7 signaling 23. Additionally, IFP in the TME can guide cell movement and promote tumor development. Research by Hyler *et al.* from Virginia Tech - Wake Forest University highlights that even low, continuous fluid shear stress can variably impact adherent epithelial ovarian cancer cells at distinct progression stages 24.

The growth and expansion of cells within the TME, especially local pressure variation, also contributed to generating indirect mechanical forces. These forces are mediated by cancer-associated fibroblasts (CAFs) and tumor-associated macrophages (TAMs). They are transmitted to mechanosensors, such as integrins, and play pivotal roles in shaping the mechanical microenvironment of tumors 25.

### Biomechanics and tumor angiogenesis

#### The effect of ECM stiffness on tumor angiogenesis

Solid stress from tumor cell growth leads to increased ECM stiffness and compositional changes 26. ECM stiffening enhances integrin-mediated Rho/ROCK activity and contraction in tumor epithelial and endothelial cells (ECs) 27. The dysregulation of mechanical force sensing contributes to aberrant behaviors in tumor ECs, resulting in abnormal structure and mechanosensitivity 27. An* in vitro* study demonstrated that elevating collagen stiffness—without altering the matrix architecture—boosted angiogenic outgrowth and increased vascular branching density in endothelial cell spheroids, thereby facilitating the formation of tumor vascular networks 28.

**Figure 2:**
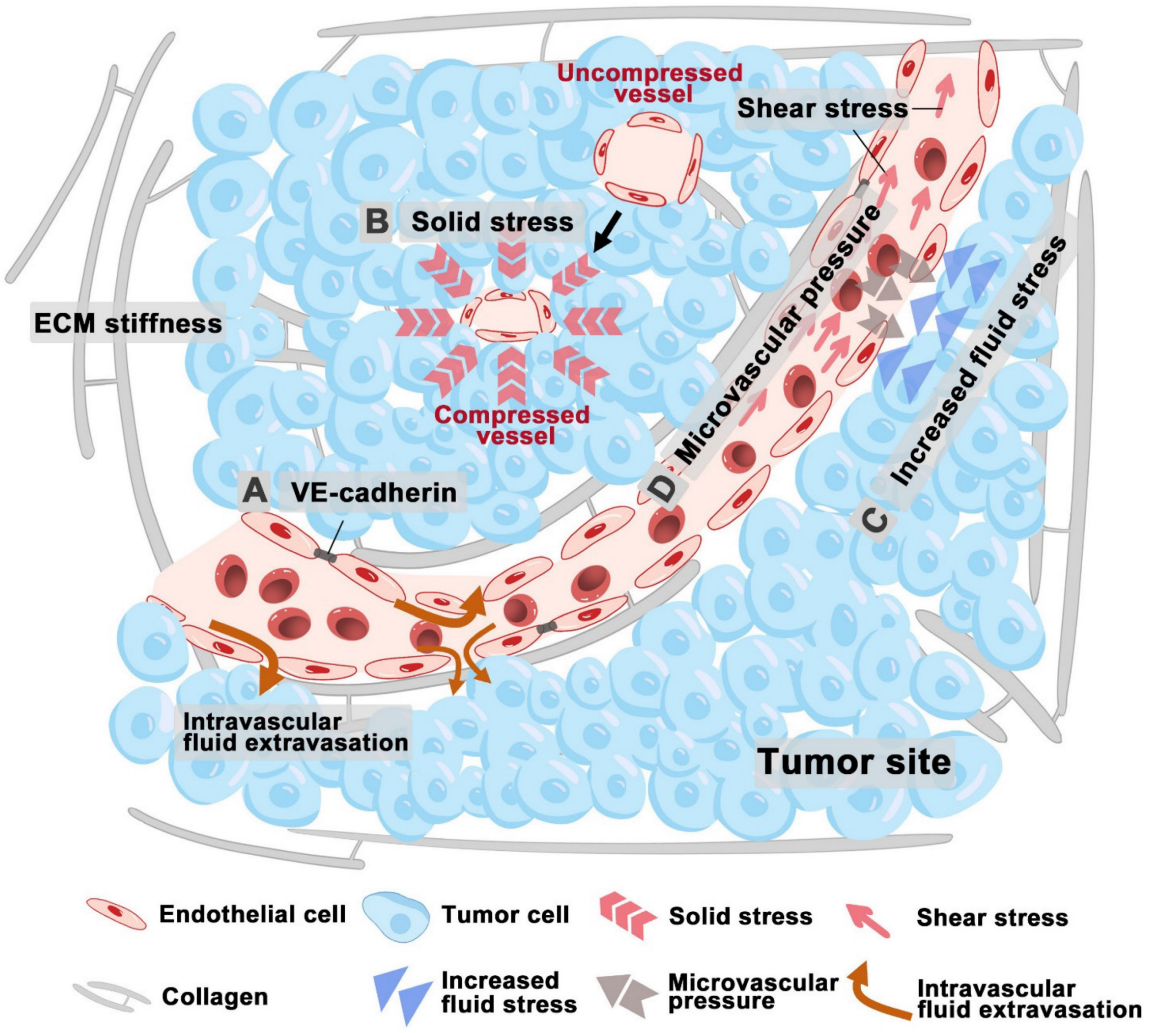
Mechanical forces within the tumor microenvironment impact tumor angiogenesis. (A) ECM stiffening alters cell-cell junctions and the positioning of VE-cadherin, thus disrupting barrier integrity and increasing permeability. (B) Solid stress compresses tumor vessels. (C) Increased fluid stress results in abnormal vascular development and inadequate tissue perfusion. (D) Elevated IFP within tumors often surpasses MVP, thereby limiting perfusion and disturbing flow patterns. Abbreviations: ECM, extracellular matrix; IFP, interstitial fluid pressure; MVP, microvascular pressure.

Increased matrix stiffness impacts the function of ECs by impairing vascular barrier integrity, altering VE-Cadherin localization, enhancing permeability, and causing morphological changes in tumor vessels (**Figure 2A**) 29. Stiffness also disrupts the expression of MS ion channels which regulate tumor angiogenesis. Moreover, the response of ECs to growth factor signaling is closely related to ECM stiffness. In hepatocellular carcinoma (HCC) cells, ECM stiffness up-regulates VEGF expression *via* the integrin β1/PI3K/Akt pathway and VEGFR2 expression in ECs through the integrin α_v_β_5_/Akt/Sp1 pathway, thus promoting angiogenesis in tumors 30, 31. Bevacizumab, a well-studied antiangiogenic agent, blocks VEGFA binding to its receptors, thereby inhibiting neovascularization and signal transduction activation 32.

#### The effect of solid stress on tumor angiogenesis

The accumulation of solid stress also impairs vascular flow in tumors by compressing the more fragile outflow vessels, such as veins and lymphatics, thereby contributing to the increased IFP (**Figure 2B**). Consequently, relieving solid stress can help decompress both blood and lymphatic vessels, leading to improved perfusion and normalization of IFP levels 33. Recent studies indicate the rising solid stress can reduce vascular patency, resulting in heightened tumor hypoxia 33, 34. This initiates a harmful feedback loop 34: tumor growth induces solid stress, which in turn causes hypoxia and prompting collagen remodeling. This remodeling affects angiogenesis and tumor cell invasion, thereby accelerating tumor progression. Solid stresses are primarily generated within matrix components, and many associated complications can be mitigated through drugs that target the degradation of these matrix elements and reduce fibrosis 33. For instance, losartan, an angiotensin receptor 1 blocker, has been shown to decrease collagen I and hyaluronic acid levels by inhibiting TGF-β signaling 35. In preclinical models of pancreatic ductal adenocarcinoma (PDAC), losartan alleviates solid stress and decompresses blood vessels, thereby improving the efficacy of chemotherapy and extending overall survival 35.

#### The effect of fluid stress on tumor angiogenesis

The abnormal blood and lymphatic vessel structures of tumor leads to increased interstitial fluid pressure and heightened permeability of blood vessels, which allows large molecules, such as plasma proteins, to cross the vascular wall and enter the tumor stroma, thereby elevating the osmotic pressure within the interstitium 36. The rapid growth of tumor cells in a confined space generates internal stress, which compresses intratumoral lymphatic vessels, thus leading to lymphatic dysfunction and fluid retention 37. Fluid stress within the TME increases viscous and geometric resistance to blood flow, thus resulting in hypoperfusion and insufficient delivery of oxygen and nutrients 29. This process ultimately results in hypoxia and a decrease in pH levels, and tumor hypoxia subsequently promotes angiogenesis 38, 39. Elevated IFP in tumors, ranging from 4 mmHg to up to 60 mmHg, facilitates the outward flow of interstitial fluid from the tumor core to its periphery.

The shear stress within tumor vessels, which is influenced by blood viscosity and shear rate, is impacted by the immature and abnormal structure of these vessels 40. The endothelial lining of the vascular network demonstrates discontinuities, lacks a complete basement membrane, and shows inadequate pericyte coverage. These structural abnormalities lead to large pores that increase blood plasma leakage into the interstitial space, thereby increasing hemoconcentration and blood viscosity 41. In tumors, the elevated IFP often surpasses microvascular pressure (MVP), which will restrict perfusion and alter flow dynamics (**Figure 2C-D**). Additionally, tumor vessels may become dilated and tortuous, potentially forming vascular shunts 42. Solid stress compresses both blood and lymphatic vessels, contributing to increased geometric resistance and significantly reducing blood flow velocity, which can be markedly lower than that in normal vessels 43, 44. This reduced shear stress in intratumoral vessels affects angiogenesis regulation and contributes to abnormalities in the tumor vascular network. Fluid shear stresses specifically influence VEGFR conformational shifts, tubule formation, and barrier integrity, ultimately directing endothelial morphogenesis and sprouting 45.

In ECs, transient receptor potential vanilloid 4 serves as a mechanosensor for both shear stress and vascular deformation, affecting tumor angiogenesis and vessel maturation. Shear stress and increased membrane tension also activate G protein-coupled receptors (GPCRs), thus triggering angiogenesis-related signaling pathways such as RhoA, PI3K, MAPK, and Akt 46. Additionally, pharmacological activation or overexpression of transient receptor potential vanilloid 4 can normalize tumor vasculature and inhibit GPCRs, thereby reducing tumor progression and enhancing the effectiveness of cancer therapies. Tyrosine kinase inhibitors like Anlotinib target these pathways, effectively suppressing angiogenesis by blocking critical phosphorylation events within ECs. Consequently, this leads to the suppression of angiogenesis. Tumors experience a combination of mechanical forces that lead to the development of dysfunctional and leaky tumor vasculature characterized by impaired barrier function and endothelial defects 47. The effective and consistent systemic delivery of cancer therapeutics remains a significant challenge in cancer treatment. To improve therapeutic delivery and efficacy, our group previously reviewed the clinical drugs aimed at normalizing tumor vasculature 48, including Sunitinib, Lenvatinib, and Nintedanib, which have been utilized in combination with chemotherapy to enhance the survival rates of cancer patients.

### Biomechanical regulation of tumor metastasis

Tumor metastasis is primarily a mechanical process 49, in which alterations in cellular biophysical properties, matrix rigidity, and the TME play crucial roles in facilitating cancer invasion and dissemination 50. The mechanical properties of cellular subcomponents are inherently associated with cancer tissues 5. For instance, in breast cancer, disruptions in the actomyosin and microtubule cytoskeletons result in a disordered network, correlating with softer and more aggressive cancer cells 51. In ovarian malignancy, reduced actomyosin contractility results in softer malignant cells, enhancing their migratory capability and aggressiveness 52. The prevailing view is that cancer cells become softer as they acquire greater aggressiveness and revert to a stiffer state when their aggressive behavior is reduced, typically through pharmacological interventions or genetic silencing of oncogenic factors across various cancers 5. However, it is important to recognize that this pattern is not universal. For instance, studies in pancreatic cancer have observed that tumors can become stiffer as they grow more aggressive due to the formation of an extensive and dense ECM 53, 54. Therefore, generalizations about changes in tumor stiffness should be made cautiously, given the considerable variability across different cancer types.

**Figure 3:**
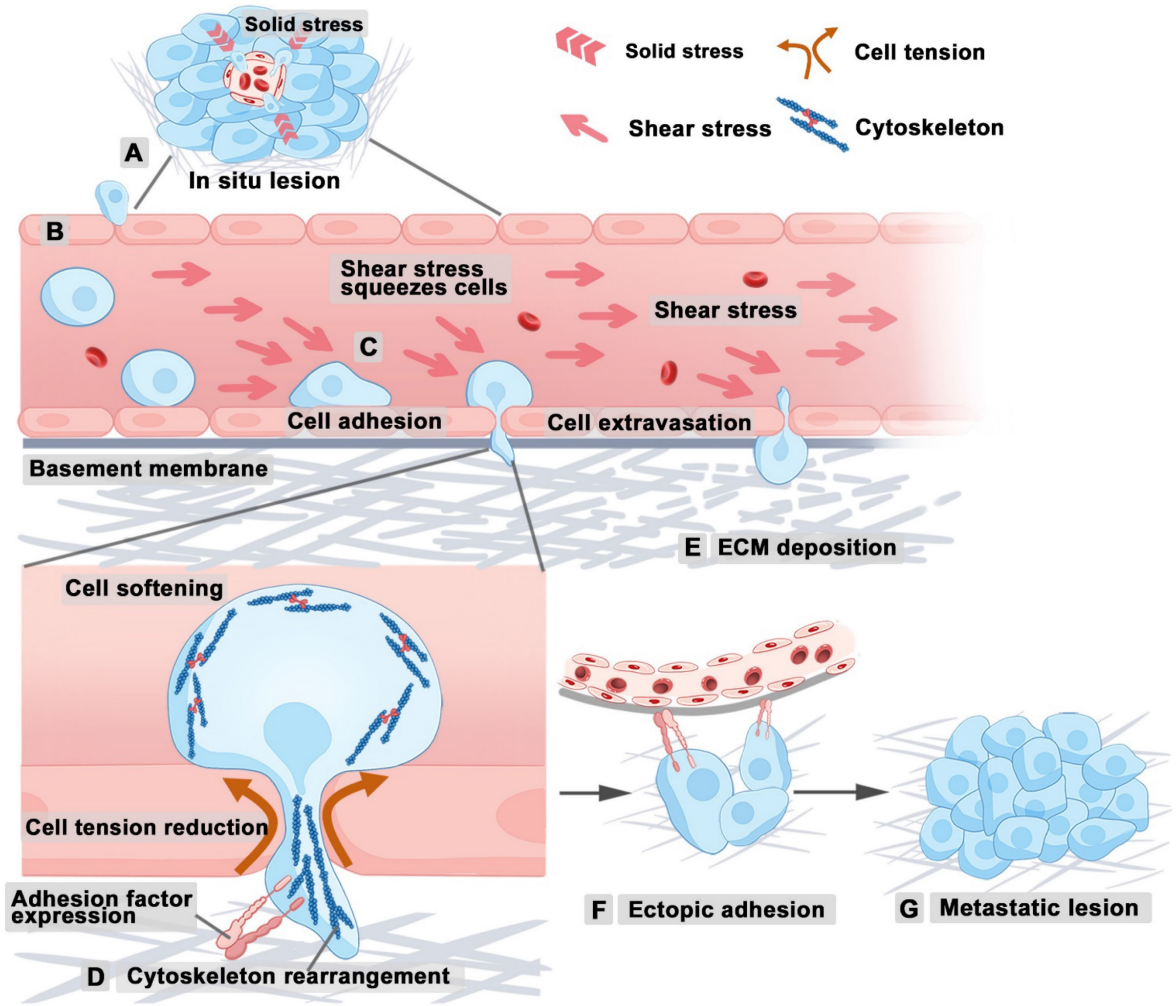
Tumor cell metastasis under biomechanical influence. (A) Tumor cells lose adhesion and detach from tumor tissue. (B) Tumor cells disrupt endothelial junctions, enabling entry into blood vessels. (C) Hydrodynamic shear stress converts CTCs into flexible cancer stem cells, enhancing their mimicry of ECs and promoting metastasis. (D) The cytoskeleton regulates tumor cell stiffness and penetration. (E) MMPs degrade the ECM, facilitating tumor cell passage through the vascular basement membrane. (F) Tumor cells adhere to blood or lymphatic vessels. (G) Metastatic tumor forms. Abbreviations: ECM, extracellular matrix; CTCs, circulating tumor cells; ECs, endothelial cells; MMPs, matrix metalloproteinases. (Adapted with permission from Ref. 209. Copyright 2024 Springer Nature)

Tumor growth intensifies solid stress due to increased cellular density and ECM deposition, thus enhancing the invasiveness of cancer cells 15. At the onset of tumor metastasis, epithelial cells undergo a transition to a mesenchymal phenotype, thereby resulting in reduced cell-cell adhesion. This process enables tumor cells to breach the basement membrane and basal lamina of the primary tumor, ultimately allowing infiltration into the surrounding tumor microenvironment (**Figure 3A**). After entering the tumor microenvironment, metastatic tumor cells sense vascular and lymphatic endothelial cells. Then, tumor cells disrupt endothelial intercellular junctions, thus facilitating their entry into blood and lymphatic vessels (**Figure 3B**), through which they spread *via* the circulatory system to distant organs. Several factors influence metastatic efficiency of tumor cells, including shear forces and vascular architecture. Hydrodynamic shear stress is known to induce the conversion of circulating tumor cells (CTCs) to less rigid cancer stem cells, enhancing their ability to mimic ECs during the metastatic processes of infiltration and extravasation, thereby facilitating tumor metastasis 2. The shear forces determine how long CTCs stay adhered to the vessel walls in larger vessels, potentially remaining dormant and increasing their chances of extravasation (**Figure 3C**) 15. Tumor cells increase intracellular pressure to facilitate nuclear passage through constrictions, such as matrix pores and intercellular gaps between endothelial cells 55, 56. During this process, the reorganization of the cytoskeleton can influence cellular stiffness and cell shape 57, thus influencing the capacity of cell to penetrate complex tumor stroma or vascular walls (**Figure 3D**). Additionally, maintaining optimal tumor cell stiffness allows the tumor cells to withstand high shear forces in the bloodstream while crossing endothelial junctions without incurring fatal nuclear damage. At the site of vascular extravasation, the MMP secreted by tumor cells can degrade ECM, reduce the solid pressure and resistance around tumor cells, and thus enable tumor cells to pass through the vascular basement membrane and move closer to the implantation site (**Figure 3E**). Upon reaching a favorable site, tumor cells adhere to the inner lining of blood or lymphatic vessels through integrin or other adhesion ligands (**Figure 3F**), thus forming secondary tumors within the lumen or extravasating through the endothelium to establish secondary growths in surrounding tissues (**Figure 3G**).

Scientists are developing therapeutic strategies aimed at inhibiting tumor metastasis through biomechanical regulation. Paclitaxel and vincristine are commonly used therapeutic drugs for tumors (including ovarian, breast, and brain tumors) in clinical practice, based on the mechanical mechanisms of stable or depolymerized microtubules (MT), indicating that the clinical application of biomechanical therapy is becoming mature 58, 59. As our understanding of biomechanical influences deepens, it is anticipated that a greater array of novel anti-tumor drugs will be integrated into clinical practice to improve the management of tumor metastasis.

### Biomechanical regulation of tumor drug resistance

Growing evidence indicates that the biomechanical microenvironment and the physical properties of tumor cells are crucial in promoting tumor resistance 60. For instance, the composition, stiffness, and structure of the ECM are critical determinants influencing the response of cancer cells to therapeutic agents 61. Adhesion of cancer cells to ECM components, such as collagen and fibronectin, or their growth in a stiff matrix, drives resistance to chemotherapy. When the ECM is stiff, ATP-binding cassette (ABC) transporters are less active and less effective at removing drugs from cells. Conversely, when the ECM is more compliant or soft, ABC transporters are more active, which can enhance drug clearance 62. Hypoxia and acidity are key characteristics of tumor metabolism that greatly enhance tumor resistance to radiation therapy, chemotherapy, and other treatment modalities 63. In the TME, hypoxia triggers stiffening of the ECM, further enhancing the drug resistance of tumor cells 64, 65.

**Figure 4:**
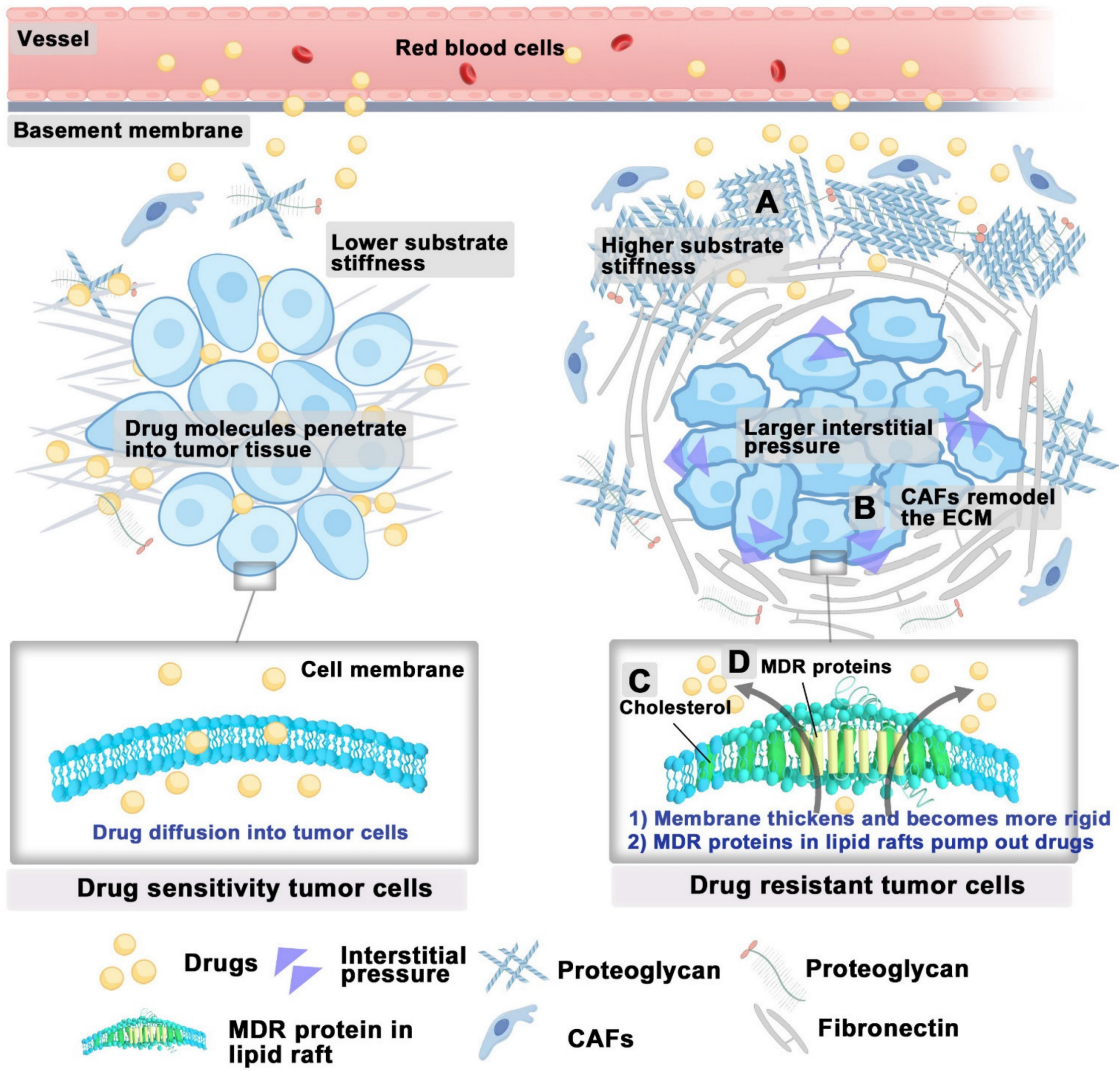
The biomechanical environment of drug-resistant tumor cells. (A) High collagen deposition in the ECM increases stiffness, contributing to drug resistance. (B) CAFs modify the ECM, promoting drug tolerance. (C) Elevated cholesterol levels in cancer cells, leading to thicker membranes that reduce drug permeability. (D) Increased cholesterol in lipid rafts enhances the function of multidrug resistance transporters, facilitating drug transport and contributing to drug resistance. Abbreviations: ECM, extracellular matrix; CAFs, cancer-associated fibroblasts.

High interstitial pressure and shear stress within the tumor can alter the morphology and behavior of tumor cells, thus enhancing the remodeling and adhesion capabilities of the cytoskeleton 5. This mechanical stress can activate multiple signaling pathways, including yes-associated protein (YAP)/transcriptional coactivator with PDZ-binding motif (TAZ), which promote the survival and resistance of tumor cells 66. Further, the stiffness of the ECM can impede drug penetration into tumors. Studies on breast cancer cells have demonstrated that their response to chemotherapeutic agents significantly varies with substrate stiffness. While the cells cultured on substrates with increased rigidity have been observed to demonstrate a heightened resistance to specific chemotherapeutic agents 67. This resistance is further supported by the high deposition of collagen proteins, which bind to proteoglycans and stabilize ECM components, thus enhancing its stiffness (**Figure 4A**). Notably, treatment with collagenase has been shown to increase IgG diffusion to tumor sites in penetration-resistant tumors 68. In all, targeting the stiffness of the ECM could offer new strategies to overcome chemoresistance.

Other evidence indicates that ECM stiffness modulates the activation of YAP, which is significantly associated with drug resistance across various human cancer cell lines 66, 69. Upon activation, the nuclear translocation of YAP may contribute to drug resistance by regulating anti-apoptotic gene transcription and interacting with the MAPK and PI3K-AKT signaling pathways 66. CAFs are primary contributors to ECM stiffness during tumor development. Within the TME, CAFs interact with cancer and immune cells, reshaping the ECM to promote tumor progression (**Figure 4B**) 70. Additionally, CAFs influence cancer cell behavior and response to treatments through ECM remodeling 71.

In addition to the tumor microenvironment, drug-resistant tumor cells exhibit distinct lipid metabolism from that of sensitive cells to reduce the damage caused by chemotherapy, thus resulting in different lipid compositions and membrane characteristics 72. For example, drug-resistant ovarian cancer cells increase the uptake of extracellular cholesterol 73, and enhance cholesterol synthesis 74, thereby elevating cholesterol levels in their membranes. The high cholesterol content in the membranes of drug-resistant ovarian cancer cells leads to thicker and more rigid membranes, resulting in reduced drug permeability, which is one of the significant reasons for the development of drug resistance in tumor cells (**Figure 4C**) 75, 76. Moreover, the increased cholesterol and sphingolipid content in the lipid rafts of drug-resistant tumor cells enhances the expression, recycling, and bioactivity of multidrug resistance transporters (such as ABC transporters) concentrated in these regions 77, 78. Cholesterol can alter the rigidity and fluidity of lipid rafts, thereby modifying the spatial conformation of multidrug resistance proteins within their domains, making it easier for these proteins to bind and transport intracellular chemotherapy drugs (**Figure 4D**) 79. Maintaining high levels of cholesterol within the lipid rafts of resistant cells is crucial for supporting the bioactivity of P-glycoprotein located therein 80. A study has shown that depleting cholesterol-enriched sphingolipid lipid rafts with small-molecule drugs can successfully reverse tumor resistance 81. In general, targeting the mechanical properties of tumor cells offers a promising strategy to overcome drug resistance.

## Decoding biomechanical signaling mechanisms of cancer cells

### Tumor cellular mechanosensors

The study of tumor cellular mechanosensors opens a crucial pathway for understanding the intricate mechanisms through which cancer cells perceive and react to biomechanical forces within their microenvironment. Tumor cellular mechanosensors primarily consist of the glycocalyx, primary cilium, cytoskeleton, and nucleus. Glycocalyx is the sugar and glycoprotein covering layer on the outside of the cell membrane. Primary cilium is a tiny protrusion on the cell membrane, and the cell membrane is the base of glycocalyx and primary cilium. The cytoskeleton is intricately linked to the cell membrane and the basal body of the primary cilium, which offers essential structural support for both. It not only senses mechanical signals but also plays an important role in transmitting these signals.

#### Glycocalyx

**Figure 5:**
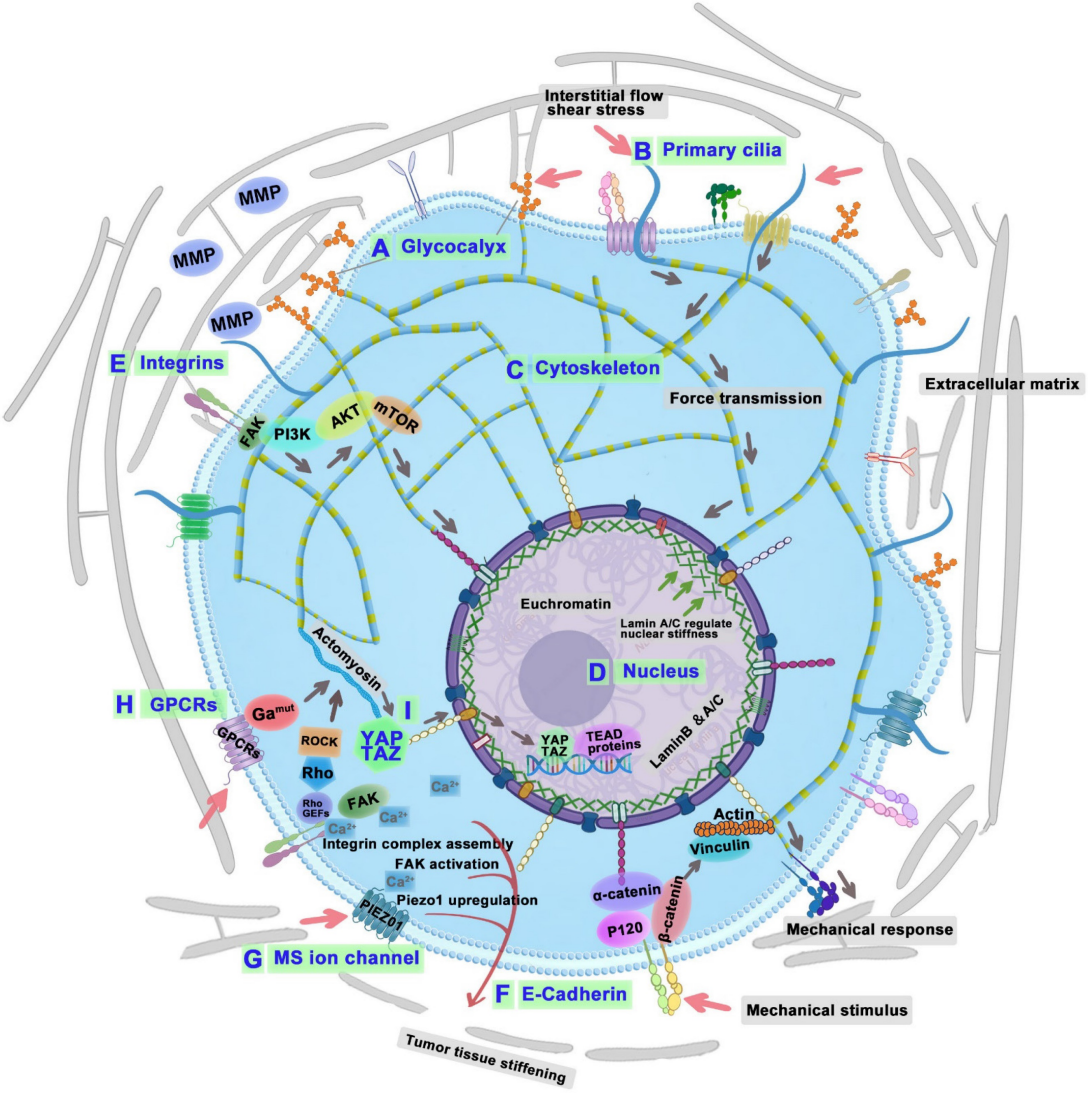
Diagram of tumor cell biomechanical perception, conduction, and effect mechanism. The GCX (A), primary cilium (B), cytoskeletonm (C), and nucleus (D) of tumor cells sense the surrounding mechanical signals; integrins (E), cadherins (F), MS ion channels (G), GPCRs (H), and YAP/TAZ (I) convert physical signals into biological signals. Decoding biomechanical signaling mechanisms of cancer cells: GCX senses shear stress and helps integrin clustering-MMP expression-tumor metastasis; PC senses fluid flow-influence cilia assembly-tumorigenesis and tumor progression; Cytoskeleton senses and transduces mechanical stresses-cytoskeletal remodeling-tumor metastasis; Nucleus senses mechanical cues-calcium channels regulation-DNA repair-tumor therapy resistance; Nucleus regulate lamin A-YAP and RAR-cytoskeleton regulation; Integrins interact with ECM components-regulates cytoskeleton-tumor metastasis; Cadherins convey mechanical signals-EGFR, catenins, and YAP-tumor proliferation, migration, and invasion; GPCRs mediate mechanotransduction-YAP signaling pathway-tumor progression and metastasis; MS ion channels convert biochemical signals-Piezo1 initiate integrin-FAK signaling-tumor invasion; TRPM7-activate EMT pathway-tumor metastasis; YAP/TAZ convert mechanical signal-matrix stiffness-tumor invasion. Abbreviations: GCX, glycocalyx; PC, primary cilia; YAP, yes-associated protein; RAR, retinoic acid receptor; ECM, extracellular matrix; EGFR, epidermal growth factor receptor; GPCRs, G protein-coupled receptors; EMT, epithelial-mesenchymal transition; MS, Mechanosensitive; TRPM7, transient receptor potential melastatin 7; YAP/TAZ, yes-associated protein/transcriptional coactivator with PDZ-binding motif.

Glycoproteins and proteoglycans represent the predominant glycan categories within the glycocalyx (GCX) 82. Proteoglycans are composed of core proteins attached to glycosaminoglycan (GAG) chains, including heparan sulfate (HS) and HA, as well as sialoglycoproteins. The GCX, interfacing directly with the ECM, plays an essential role in mediating integrin adhesions to the ECM and in responding mechanically to environmental stiffness (**Figure 5A**) 83. Furthermore, the specific composition and size of the GCX influence the extent of mechanosensing experienced by cell-bound integrins upon contacting the ECM 84. Notably, bulky cancer-associated glycoproteins like MUC1 are known to facilitate integrin clustering and enhance mechanosensing capabilities 85. Research indicated that overexpressing MUC13 in Panc-1 cells typically reduced their modulus and diminishes adhesion. Conversely, knocking down MUC13 in HPAF-II cells leads to increased modulus and enhanced adhesion 86. Therefore, it is speculated that tumor cell-cell adhesion can be enhanced and invasiveness can be reduced by reducing the volume or directly knockdown of the expression of GCX.

The GCX on cancer cells is notably dense, aiding in integrin clustering, growth factor signaling, and mechanotransduction of elevated interstitial flow shear stress within tumors. This process subsequently promotes release of MMPs, which will enhance cell motility and metastasis 82. Research by Qazi *et al*. from City University of New York indicated that such interstitial flow notably increased migration in SN12L1 cells (high metastatic potential) of human kidney carcinoma lines, unlike in SN12C cells (low metastatic potential) 87. Specifically, the expression of MMP-1, MMP-2, CD44, and α3 integrin were upregulated by interstitial flow in SN12L1 cells, while it remained unchanged in SN12C cells. Moreover, enzymatic cleavage of GCX components, such as HS or HA, inhibited flow-induced migration and MMP expression in SN12L1 cells. This suggests that the GCX in cancer cells serves as a mechanosensor for interstitial flow shear stress, coordinating the expression of MMP-1, MMP-2, CD44, and α3 integrin to control cell migration and metastasis. Additionally, 4-Methylumbelliferone inhibits HA synthesis by downregulating HA receptors and the phosphatidylinositol 3-kinase/CD44 complex 88. The anti-CD44 monoclonal antibody A6 has been shown to inhibit tumor cell migration, invasion, and metastasis by blocking CD44-mediated signaling pathways 89.

Understanding the MS and transductive functions of the GCX on tumor cells have paved the way for innovative cancer therapeutic strategies. First, modulating GCX mechanotransduction will block GCX-mediated adhesive interactions, which will reduce tumor cell extravasation, potentially halting metastasis and improving patient survival rates 90. Second, reducing the thickness of the GCX enhances immune recognition by natural killer cells, which can be achieved by degrading the GCX, thereby augmenting the cytotoxicity of these immune cells 91. Lastly, editing the composition of the GCX through self-executed feedback loops presents a novel and manageable approach to cancer treatment 92.

#### Primary cilia

Primary cilia (PC) consist of a microtubule-based core, called the axoneme, which extends from a specialized centriole known as the basal body and is enclosed by a lipid bilayer continuous with the cell membrane (**Figure 5B**). Despite their small size, PC constitutes approximately 1/200 total surface area of the cell. The PC are critical for both development and homeostasis of the body. These structures are densely packed with receptors, ion channels, and downstream signaling molecules critical for numerous pathways, such as Hedgehog and GPCR signaling. The absence of this antenna-like structure results in improper signaling activation. Consequently, mutations that disrupt the assembly, structure, or function of cilia impair the transmission of mechanical signals, resulting in ciliopathies—a diverse group of over 30 human diseases and syndromes affecting various organs and tissues, including the eye, heart, kidney, brain, liver, and bone 93.

Under fluid flow stimulation, PC deflect, transmitting mechanical strains *via* the cytoskeleton to critical cytoplasmic organelles like the Golgi complex, which governs the response of cell to mechanical stimuli. Modifying the length and rigidity of PC can influence this cellular mechanosensitivity 94. Notably, primary cilia are frequently absent in various cancers 95, including glioblastoma, melanoma, pancreatic, prostate, ovarian, colon, breast, medulloblastoma, and renal cancers, as opposed to their presence in normal tissue 96. In cholangiocarcinoma cases without primary cilia, inhibiting histone deacetylase 6, a protein involved in cilia disassembly, has been shown to restore cilia formation and suppress tumor growth 97.

Approximately 25% of tumors in patients with PDAC exhibit PC. The presence of PC is associated with an increased incidence of lymph node metastasis 98. Research by Martínez-Hernández et al. from Spain demonstrated a marked elevation in PC levels in pituitary neuroendocrine tumors (PitNETs), which was associated with increased tumor invasiveness and higher recurrence rates^99^. Additionally, molecular analysis revealed the dysregulation of 123 cilia-associated genes, including doublecortin domain containing protein 2, syntaxin-3, and centriolar coiled-coil protein 110 in PitNETs. Moreover, an increase in both the formation and length of primary cilia has been observed in cancer cells that exhibit resistance to anti-cancer drug kinase inhibitors 100. Thus, regarding a clear link between PC and tumorigenesis, the impact of PC on cancer progression may differ depending on the specific type and stage of the cancer. Regulating the expression and mechanical properties of PC holds the potential to unveil new therapeutic strategies, given their pivotal role in biomechanical signal transduction and resistance to chemotherapy in cancer cells. Future investigations might focus on accurately modulating these structures to enhance therapeutic outcomes and curtail tumor progression.

#### Cytoskeleton

The primary components of cytoskeleton include MTs, actin filaments, and intermediate filaments. It not only senses and transduces mechanical stress but is also influenced by external forces from the ECM (**Figure 5C**) 101. When mechanical forces are applied to cancer cells, actin filaments act as mechanosensors that detect these forces 102. These filaments generate contractile forces through interactions with myosin II and through polymerization, which drives the forward movement of the plasma membrane 103. MTs are essential in aligning chromosomes and organizing the spindle in response to mechanical forces during mitosis 104. In tumor cell migration, MTs facilitate pseudopodia formation, which reacts to mechanical signals from the TME 105. Intermediate filaments, recognized for their stability and durability, are critical in sensing the magnitude and direction of mechanical forces encountered by cancer cells. As tumors progress, the cytoskeleton undergoes continual remodeling, allowing tumor cells to develop distinctive mechanical properties and adapt to the dynamic shifts within their microenvironment 106. During tumor progression, tumor cells actively remodel their cytoskeletal structures and decrease cellular stiffness 107. As tumor cells enter and exit the vascular system, they experience significant shape alterations facilitated by cytoskeletal remodeling, which enable them to traverse endothelial cell-cell junctions 108. Research by Liu *et al*. from Chengdu Medical College has shown that low shear stress markedly enhances both the percentage and length of filopodia, which are vital for cancer cell mobility and can trigger migration^109^. However, shear stress may also influence tumor progression through synergistic interactions with chemical factors like chemokines or growth factors, and mechanical factors such as matrix stiffness. Further research is needed to elucidate the complex tumor microenvironment's impact. Recent findings indicate that the cytoskeletal structure and biophysical characteristics of breast cancer subgroups are linked to their metastatic preference, regarding the gene expression profiles and mechanoadaptation capacities 110. Therefore, by increasing the shear stress and inhibiting Cdc42, filopodia is greatly reduced, thereby reducing tumor metastasis.

Modulating the mechanical properties of the cytoskeleton is a promising strategy for tumor therapy. A research obstacle is to develop equipments capable of measuring and applying forces. Future studies should focus on integrating mechanotransduction research with therapeutic interventions by identifying key molecules that promote cell health or treat diseased cells. Additionally, it is important to understand how cellular mechanosensors interact with the tumor microenvironment to activate cytoskeletal movements. This will require a multidisciplinary approach to model mechano-responses and develop treatments that can reverse cancer pathologies.

#### Cell nucleus

The nucleus, notable for being both the largest and stiffest organelle, is also highly dynamic, capable of sensing external mechanical cues and adapting rapidly 111, 112. The nucleus plays an integral role in mechanoregulation, which encompasses both mechanosensing and mechanotransduction processes (**Figure 5D**). Surface mechanoreceptors detect these cues and transmit signals to the nucleus, influencing cytoskeletal integrity and tension. This leads to adjustments in gene expression related to mechanical stimulation 113. Changes in nuclear mechanics, such as those induced by the ECM, can influence the morphology of nucleus and localization of transcription factors 114. Cellular adaptations to matrix tension involve alterations in lamin A phosphorylation and nuclear positioning, which are regulated *via* the mechanotransduction pathways of YAP and retinoic acid receptor (RAR), ensuring cytoskeletal equilibrium 45. Cells adapt to matrix tension by modifying lamin A phosphorylation and nuclear positioning, and maintain cytoskeletal balance through the mechanosignaling routes of YAP and RAR 114.

Cell spreading and nuclear stretching activate MS calcium channels on the nuclear membrane, leading to an increase in nuclear calcium levels. This increase causes elevated levels of the transcription factor CREB, which is vital for regulating gene transcription, protein import, apoptosis, and subsequent mechanosignaling processes 115, 116. The phosphorylation of Lamin A/C and Emerin within the nucleus responds to mechanical stimulation by altering nuclear stiffness and nucleo-cytoskeletal coupling 114. Further, changes in chromatin organization, condensation, and modification are influenced by the actin cytoskeleton and the linker of nucleoskeleton and cytoskeleton complex 117, 118.

Cytoskeletal contraction also triggers adenosine triphosphate (ATP) release and calcium signaling, which facilitate the nuclear import and activation of histone modifiers, such as enhancer of zeste homolog 2 and histone deacetylase 117, 118. These processes drive cancer-related gene silencing and transcriptional regulation through alterations in histone methylation 119 and acetylation 120. Furthermore, polymerization of nuclear actin adjusts nuclear structure and transcription factor functionality, influencing gene expression through enhanced nuclear transport mechanisms 121. Softer nuclei, characterized by reduced levels of lamin A/C, are more susceptible to rupture and subsequent DNA damage during migration 122. In contrast, cells with stiffer nuclei, induced by progerin, also exhibit increased DNA damage 123. This paradox highlights the complex role of nuclear mechanics in cellular health. The research conducted by Nava *et al.* from University of Helsinki demonstrated that mechanical stretching of the nucleus induced a calcium-dependent softening mediated by chromatin alterations, and inability to initiate the nuclear MS response led to DNA damage 124. Subsequent DNA damage response reorganizes the nucleus, altering chromatin structure to facilitate more efficient DNA repair, which may inadvertently contribute to chemotherapeutic resistance 125.

### Mechanosignaling proteins

In addition to cellular mechanosensors, a range of mechanical signals are perceived and relayed to cells *via* the activation of surface mechanosignaling proteins like integrins 83, YAP/TAZ 126, transient receptor potential (TRP) ion channels 127, GPCRs 128, and Piezo channels 129. The mechanosignaling proteins transmit these cues to cellular internal components, thus influencing the behavior of tumor cells.

#### Integrins

Integrins, which are transmembrane proteins, bind to diverse ECM proteins and play a pivotal role in detecting changes in the extracellular environment (**Figure 5E**). These proteins are critical for cell adhesion and signal transduction. They facilitate the detection of the mechanical properties within the ECM and relay these signals to focal adhesion kinase (FAK). This interaction strengthens focal adhesions and triggers subsequent intracellular signaling pathways 130. In the TME, FAK influences both cancer and stromal cells, enhancing tumor progression and metastatic potential 131.

Integrin-mediated adhesions engage with the ECM and respond to its rigidity, consequently influencing cellular activities including motility and migration 132. Integrin interactions with specific ECM components trigger outside-in signaling that regulates the cytoskeleton. Concurrently, mechanical forces generated by the cytoskeleton are transmitted back to integrin-ECM interactions, promoting cancer metastasis 133. Several clinical studies have linked high integrin expression to poor cancer survival 106. Integrin-mediated mechanotransduction reciprocally affects the mechanical properties of the TME. In non-small cell lung carcinoma cells, the absence of integrin α11 is associated with reduced collagen reorganization and lower tissue stiffness, which in turn inhibits cell growth and metastatic potential 134. This phenomenon highlights the pivotal role of stromal integrin α11 expression in collagen cross-linking. In colon cancer cells, integrins are responsive to mechanical stimuli, particularly shear stress, which leads to the downregulation of integrin β1-FAK signaling, subsequently enhancing the cytotoxic effects of radiation 135. The deregulation of integrin signaling, facilitated by alterations in the ECM and integrin diversity, allows cancer cells to rapid cell proliferation, invade tissues, and adapt to different environments 136. As a result of dynamic remodeling of the ECM, tumor cells change in density, hardness, or tissue composition. For instance, the progression of breast cancer is associated with elevated mechanosignaling and increased tissue birefringence, suggesting that ECM hardness promotes malignancy and increases tumor aggressiveness 137. Moreover, hypoxia-inducible factor 1 upregulates the expression of lysyl oxidase which enhances the crosslinking of collagen fibers. This process increases the stiffness of the TME, which in turn enhances integrin-mediated signaling and promotes cell proliferation 138.

During the early stages of tumorigenesis, neoplastic conversion significantly impacts the expression levels of specific integrins, resulting in changes to the integrin profile on cancer cells. It triggers modifications in integrin signaling pathways that facilitate the advancement of neoplastic transformation 139. Oncogenic signaling plays an important role in driving these alterations. For example, in terms of ovarian cancer, mutant p53 operates via integrin α_5_β_1_ to enhance the expression of the epithelial-mesenchymal transition (EMT) transcription factor TWIST1. This process promotes the formation of tumor cell clusters that penetrate the mesothelium and subsequently proliferate into peritoneal tumors 140. However, certain integrins, like α_2_β_1_, may impede tumor progression, highlighting the complex and variable roles of integrins in cancer 141. Given their overlapping functions in adhesion and signaling, it is challenging to develop specific inhibitors and sensitive biomarkers. Over the past 30 years, many drug discovery projects and clinical studies have focused on integrins. However, the approved anti-cancer drugs targeting integrins are limited 142. Therefore, a comprehensive investigation into integrin dependency across various cancer types, coupled with biomarker development using genetically engineered and patient-derived xenograft models, is essential for advancing integrin-targeted cancer therapies.

#### Cadherins

Cadherins, such as E-, VE-, N-, R-, P-, and K-cadherin, are transmembrane proteins that function as cell-cell interaction receptors and enable calcium-dependent adhesion 143. In tumors, cadherins act as critical mechanosensors that detect and convey mechanical signals from neighboring cells (**Figure 5F**). The cadherin cytodomain connects to the actin cytoskeleton through β-catenin and α-catenin, thereby regulating mechanotransduction 144. Among the classical family of cadherins, E-cadherin plays a central role as a mechanosensor by both sensing and facilitating the transmission of mechanical forces 145. The force transduction mediated by E-cadherin influences various cellular functions. It activates signaling *via* the epidermal growth factor receptor (EGFR), which governs local cytoskeletal restructuring and promotes cellular proliferation 146. E-cadherin is identified as a tumor suppressor protein, and its decreased expression associated with the EMT is a common occurrence in the process of tumor metastasis. By enhancing E-cadherin expression, α-solanine (a glycoalkaloid extract of Solanum nigrum Linn) inhibited endothelial cell transformation and exhibited potent anti-carcinogenic properties 147.

Moreover, the internalization of E-cadherin in response to blood flow may represent an adaptive metastatic mechanism that enhances cellular motility and invasion 148. Concurrently, a stiffer ECM elevates N-cadherin expression on endothelial cells, enhancing their interaction with tumor cells and vascular endothelium to facilitate metastasis 149. Additionally, E-cadherin affects the activity of transcriptional coregulators such as catenins and YAP. Under biaxial mechanical stretch conditions, YAP and β-catenin, which are components of the cadherin complex, promote cell cycle progression in an E-cadherin-dependent manner 150. The modulation of actin cytoskeleton rigidity influences the interaction between APC and β-catenin, thereby affecting the localization of β-catenin within the nucleus or cytoplasm. The suppression of β-catenin-mediated transcription impeded the progression of the cell cycle from the G1 phase to the S phase 150. Moreover, the cadherin-mediated mechanical force transmission, especially *via* the N/E-cadherin complex, is key to tumor cell migration and invasion. Inhibiting this complex can reduce interactions between mesenchymal-like and epithelial-like cancer cells, thus decreasing tumor aggressiveness 151. Overall, the cell-cell interaction mediated by cadherin is crucial for the migration, survival, and proliferation of cancer cells. However, the specific impact of cadherin-driven mechanotransduction on tumor progression *in vivo* warrants further exploration.

#### Mechanosensitive ion channels

During tumor progression, mechanical cues activated by MS ion channels influence both the cancer cells and their surrounding microenvironment. These mechanical signals are converted into cellular responses, including proliferation (**Figure 5G**) 152. MS ion channels, including epithelial sodium channels, TRP channels, two-pore domain potassium channels, and Piezo channels, convert mechanical stimuli at the cell membrane into biochemical signals *via* mechanotransduction 5, 153.

Piezo1 and Piezo2, the primary mechanosensors in mammals, facilitate cellular adaptations to mechanical forces 153. Their upregulation is linked to increased proliferation, migration, and invasion in tumor cells, suggesting their potential as therapeutic targets in cancer 154, 155. Changes in the matrix microenvironment may result in the overexpression of certain MS ion channels, including Piezo1. Specifically, the study by Chen *et al*. from hospital for sick children in Canada demonstrated that Piezo1 activation could initiate integrin-FAK signaling, influence ECM composition, and contribute to tissue stiffening. Meanwhile, the stiffer environment increased the expression of Piezo1, inducing glioma aggression 156. Thus, targeting Piezo1 offers a potential strategy to interrupt the harmful feedback loop between the mechanotransduction of tumor and abnormal tissue mechanics 156. Mechanical stimuli, including stretch and compression, activate Piezo1 and its associated signaling pathways, such as the Akt/mTOR pathway in prostate cancer 106, thereby promoting cell cycle progression and enhancing tumor cell invasion as well as matrix degradation. Additionally, Piezo1-mediated calcium influx induced by circulatory shear stress increases susceptibility of cancer cells towards TRAIL-induced apoptosis 157, underscoring the potential of targeting Piezo1/2 in cancer therapy.

As cationic channels, TRP proteins could be activated by physicochemical stimuli to regulate diverse sensory capabilities which are associated with various cancers 158. Among them, transient receptor potential melastatin 7 (TRPM7) is a MS TRP ion channel, whose expression is notably altered in various cancers 159. High TRPM7 levels are linked to EMT pathway activation and are associated with reduced disease-free and overall survival in ovarian cancer cells 160. Additionally, TRPM7 is essential for activating Notch and JAK/STAT3 pathways in glioblastoma, increasing the levels of cancer stem cell marker ALDH1 159. Above all, these insights underscore the potential of MS ion channels as key targets in the development of novel cancer therapies, leveraging their MS properties to counteract tumor progression.

#### G protein-coupled receptors

GPCRs constitute the largest family of membrane receptors, characterized by diverse intracellular signaling properties that originate from the activity of G-protein subunits 161. GPCRs have been postulated independently to mediate mechanotransduction 162 and to facilitate changes in cell shape (**Figure 5H**) 163. Recently, the function of GPCRs as mechanosensors in cancer cells has been progressively demonstrated 106. Yang *et al*. 164 from first affiliated hospital of Xi'an Jiaotong University showed a member of the GPCR family, C-X-C chemokine receptor type 4 (CXCR4), acted as a crucial intracellular signal transducer to regulate mechano-sensitive cellular activities through YAP signaling pathway mediated by ubiquitin domain-containing protein 1. Their study demonstrated that the expression of CXCR4 was significantly upregulated in HCC cells as matrix stiffness increased, driving cell growth, EMT, and cancer cell stemness. Notably, luteolin, a natural compound, was found to suppress the effects induced by matrix stiffness and block the CXCR4-driven YAP signaling pathway within HCC cells 164.

Evidence suggested that targeting GPCR function could effectively slow or prevent the progression and metastasis of various cancers 165. GPCRs, such as those responsive to chemokines, thrombin, and neuropeptides, represent promising targets for pharmacological interventions in cancer prevention and therapy 165. Studies by Liu *et al*. from Kunming University of Science and Technology have demonstrated that GRPR-specific inhibitors could significantly reduce tumor growth and angiogenesis, highlighting their potential in clinical cancer management 166. Despite GPCRs being crucial drug targets, their exploitation as cancer targets is limited, with few anti-cancer compounds that modulate GPCR-mediated signaling currently in clinical use 166. Maraviroc, an FDA-approved antagonist of the C-C chemokine receptor 5 (CCR5), showcases the potential of small molecules in inhibiting GPCRs 167, 168. A phase I trial confirmed the anti-tumor effects of a CCR5 antagonist in patients with advanced, refractory colorectal cancer and liver metastases 169. In general, GPCRs play a crucial role in transducing mechanical signals within tumor cells. Moreover, combinatorial immunotherapies that target GPCRs are emerging with promising effects for cancer treatment, highlighting the potential of GPCRs in mechanotransduction and cancer cell behavior 170.

#### YAP/TAZ

YAP and the transcriptional coactivator TAZ function as mechanosensors and mechanotransducers, responding to ECM stiffness, cell morphology, and cytoskeletal tension, which are essential for nuclear localization (**Figure 5I**) 171. YAP/TAZ activity is closely linked to the structure of the actin cytoskeleton, which reinforces membrane-cytoskeleton integrity and supports cancer cell viability during metastasis 172. These proteins are central to tumor morphogenesis by reshaping the TME to promote growth and evade immune surveillance, influencing not only tumor cells but also surrounding fibroblasts, immune, and endothelial cells 69.

YAP/TAZ are frequently deregulated in cancer due to alterations in mechanotransduction, inflammation, oncogenic signaling, and inhibition of the Hippo pathway 173. This deregulation enhances force transmission between oncogene-expressing cells and the ECM, facilitating tumorigenesis through YAP/TAZ mechanotransduction 174. YAP additionally stimulates the expression of cytoskeletal regulators, which allows fibroblasts to increase matrix stiffness and facilitate cancer cell invasion 175.

Targeting YAP/TAZ could be a viable cancer treatment strategy. IAG933, an inhibitor developed by Novartis targeting YAP/TAZ-mediated transcription, is currently undergoing a phase I clinical trial for tumors with YAP/TAZ gene fusions (NCT04857372) 69. Similarly, VT3989 from Vivace Therapeutics is undergoing a Phase I trial for solid tumors and mesotheliomas with NF2 mutations (NCT04665206) 69. Drugs like dasatinib, targeting SRC family members, also show potential in inhibiting YAP/TAZ activity in both laboratory and clinical settings 176. However, the clinical efficacy of these treatments has been variable, indicating a need for continued research into effective YAP/TAZ inhibitors 176. While research is still in its early stages, these studies are expected to be crucial for developing new anti-tumor drugs and treatment strategies in the future.

#### Other mechanosignaling proteins

Rho GTPases, a family of small G proteins, are essential regulators of cytoskeletal dynamics, cell polarity, motility, vesicular transport, cell cycle progression, differentiation, and gene expression 177. Activation of growth factor receptors and integrins promotes the exchange of guanosine diphosphate (GDP) for guanosine triphosphate (GTP) on Rho proteins, allowing GTP-bound Rho proteins to interact with effectors that regulate their activity and localization 178. In humans, around 20 kinds of Rho GTPases have been identified, with RhoA, Rac, and Cdc42 being the most extensively studied. These proteins are key in remodeling actin-rich cytoskeletal structures and regulating cell contractility, influencing many cellular processes 179. In cancer, Rho GTPases are typically overexpressed 177. The overexpression of active RhoA, RAC1 180, or Cdc42 181 in rodent fibroblasts enhances anchorage-independent growth and tumorigenesis. Furthermore, effectors such as Rho-associated coiled-coil-containing protein kinase (ROCK) and p21-activated kinases (PAKs) play an important role in cellular transformation; elevated levels of ROCK2 have been associated with high-risk neuroblastoma and adverse patient outcomes, indicating that ROCK inhibitors could offer therapeutic benefits 182. Targeting these regulators, either alone or combined with MAPK or SRC therapies, may offer effective treatment options. Recently, small-molecule inhibitors of Rho GTPases have shown promise *in vitro* and *in vivo* 183. For example, AZA1, a specific inhibitor of Cdc42/RAC1, effectively suppresses prostate cancer growth *in vivo* and improves survival in mouse models 184.

Research has demonstrated that forces applied to the cell surface can transmit to chromatin *via* the cytoskeleton and nuclear proteins, leading to chromatin stretching and activation of gene expression 15. Nuclear proteins primarily regulate gene expression, translation, and related processes 185. Abnormal expression of certain nuclear proteins is associated with tumorigenesis, drug resistance, and metastasis 186, 187. Notably, mutations in these proteins can affect nuclear mechanics and cytoskeletal organization, influencing various cellular functions 188. For example, mutations in nuclear envelope proteins disrupt mechanotransduction signaling and force transmission 189. Poh *et al*. from University of Illinois at Urbana-Champaign 190 found that applying excessive force led to rapid and irreversible dissociation of survival of motor neuron from coilin in the Cajal body of HeLa nuclei 190. This dissociation was sensitive to substrate stiffness, suggesting that sufficient cytoskeletal tension is essential for transmitting forces to the nucleus and inducing deformations 191. Since Cajal body interact directly with chromatin, these results indicate that force-induced dissociation of nuclear proteins can alter gene expression. Further studies are necessary to determine the functional consequences and longevity of these transcriptional changes 192.

In general, mechanosignaling proteins are being recognized for their pivotal roles in the occurrence and progression of tumors 5. Additionally, these proteins that influence the mechanosensitivity and mechanotransduction of cancer cells represent potential therapeutic targets. Numerous agents that block mechanosignal transduction have already entered clinical trials (**Table 1**). As research advances, biomechanical regulation strategies are expected to pioneer new avenues for cancer therapy.

## Biomechanical regulation tumor nanotherapeutic strategies

Nanotechnologies offer transformative potential in biomechanical regulation for tumor therapy by targeting the mechanical characteristics of TME and cancer cells. Such technologies enable precise control over cellular biomechanics, which is crucial for developing effective therapies. For example, the unique enhanced permeability and retention (EPR) effect of tumor tissue can retain more nano-sized systems, thereby achieving passive drug enrichment in the tumor site; targeted drug delivery systems can selectively interact with primary cilia or cytoskeletal components, thus enhancing the therapeutic efficacy on tumor; advances in molecular self-assembly technologies and mechanical modulation of the ECM hold promise for disrupting tumor progression and improving treatment outcomes 193. In this context, we delineate innovative therapeutic approaches leveraging nanotechnology to modulate the perception and transduction of tumor biomechanical signals. These strategies are designated as biomechanical regulation tumor nanotherapeutic strategies (**Table 2**).

### Interfering primary ciliary biomechanical function

Primary cilia play a role in sensing chemical and mechanical signals. Compounds that regulate cilia length can enhance mechanosensitivity 194. In glioblastoma, primary cilia formation is reduced. Loskutov et al. from Virginia University School of Medicine 195 reported that lysophosphatidic acid receptor 1 (LPAR1) accumulates in cilia, where it binds lysophosphatidic acid (LPA) to promote cell proliferation. When cilia are lost, LPAR1 moves to the plasma membrane, driving tumor cell proliferation. The small molecule Ki16425 inhibits LPA signaling and suppresses glioblastoma growth. In a mouse model, Ki16425-loaded nanoplatforms significantly reduced tumor progression, suggesting a potential therapeutic strategy for glioblastoma.

**Figure 6:**
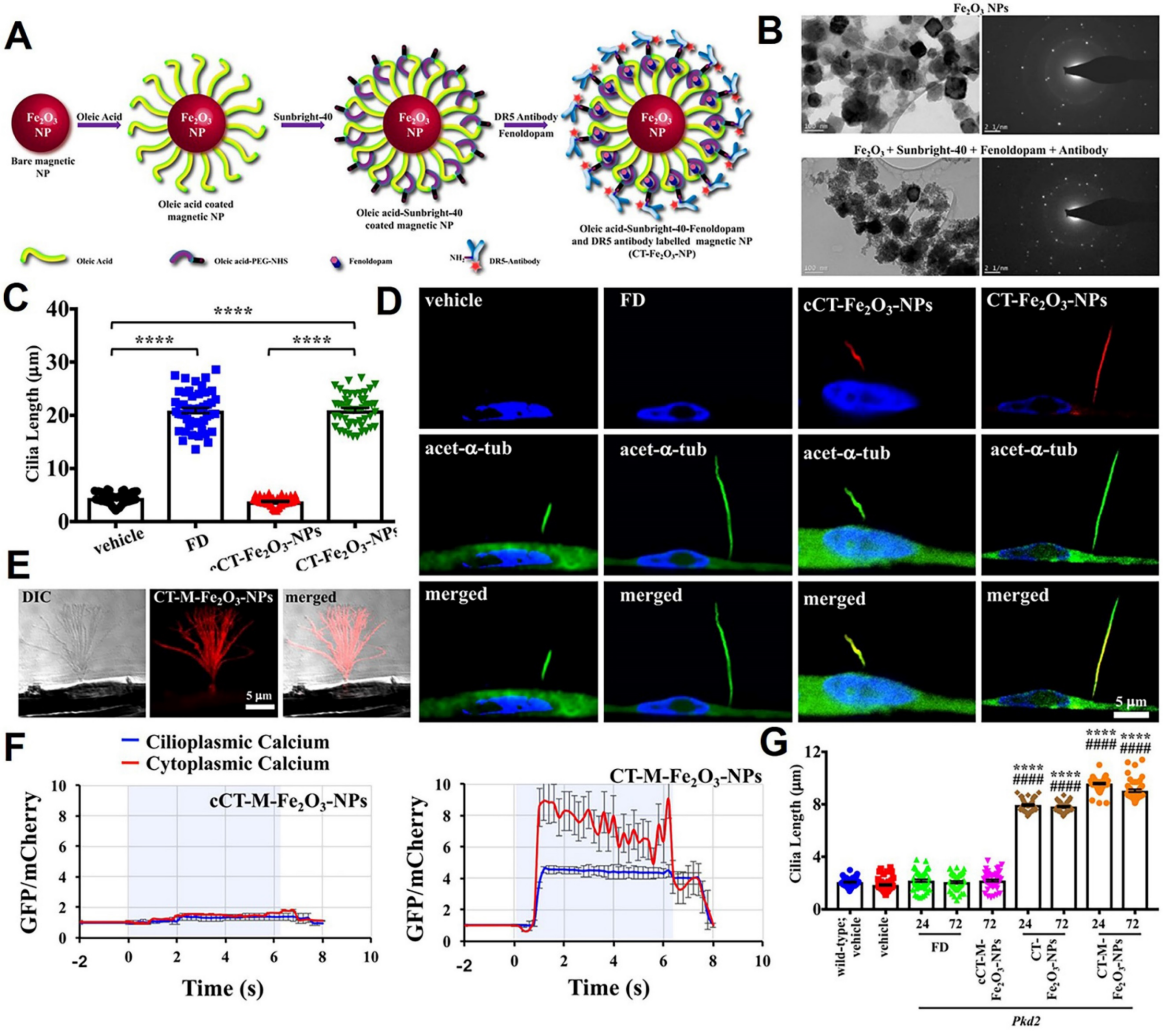
Design nanoplatforms for interfering with the biomechanical function of primary cilia. (A) Synthesis and surface functionalization of CT-Fe_2_O_3_-NPs. (B) TEM and selected area electron diffraction images of bare Fe_2_O_3_-NPs and CT-M-Fe_2_O_3_-NPs. (C) A representative dot-plotted bar graph displaying the ciliary lengths measured in cells subjected to various treatments. (D) Fluorescence images illustrating that both fenoldopam and CT-Fe_2_O_3_-NPs (red) resulted in increased cilia length. (E) An external magnetic field applied to CT-M-Fe_2_O_3_-NPs induced passive movements of the cilia. (F) Line graphs depicting average cytosolic (red) and cilioplasmic (blue) Ca^2+^ levels (in arbitrary units). (G) Dot-plotted bar graphs showing cilia lengths in vascular endothelial cells. (Adapted with permission from Ref. 196. Copyright 2019 American Chemical Society)

Primary cilia are cell organelles that expose themselves to the extracellular lumen, providing an important access to target the cilia. With a diameter of about 250 nm, primary cilia make nano-sized particles promising vehicles for drug delivery. In a study, Pala *et al*. from University of California Irvine reported a kind of cilia-targeted (CT) nanoparticles for the precise delivery of the therapeutic drug (fenoldopam), termed CT-Fe_2_O_3_-NPs (**Figure 6A-B**) 196. High-resolution differential interference contrast imaging was used to locate cilia and assess the selectivity and specificity of CT-Fe_2_O_3_-NPs. Results indicated that both control CT-Fe_2_O_3_-NPs without fenoldopam (cCT-Fe_2_O_3_-NPs) and CT-Fe_2_O_3_-NPs exhibited specific CT delivery; however, only CT-Fe_2_O_3_-NPs containing fenoldopam significantly increased cilia length (**Figure 6C-D**). Notably, CT-Fe_2_O_3_-NPs also enabled remote manipulation of cilia movement and function *via* an external magnetic field (**Figure 6E**). Cilia function was assessed by monitoring changes in cytosolic Ca^2+^ concentrations. Application of a magnetic field caused significant cilia bending and a sustained rise in Ca^2+^ signaling within both the cilioplasm and cytoplasm in cells treated with cilia-targeted nanoplatforms, compared to controls (**Figure 6F**). In the *in vivo* study, localization of CT-Fe_2_O_3_-NPs in the vascular endothelium was confirmed at 24 h and 72 h post-injection. Cilia length was notably increased in mice treated with CT-Fe_2_O_3_-NPs or CT-M-Fe_2_O_3_-NPs (under magnetic field exposure) but not in those receiving a 30-min fenoldopam infusion (**Figure 6G**). The results in this section demonstrated that controlling ciliary movement to block the conduction of mechanical signals can achieve efficient tumor treatment.

### Interfering protein biomechanical sensing-transduction function

**Figure 7:**
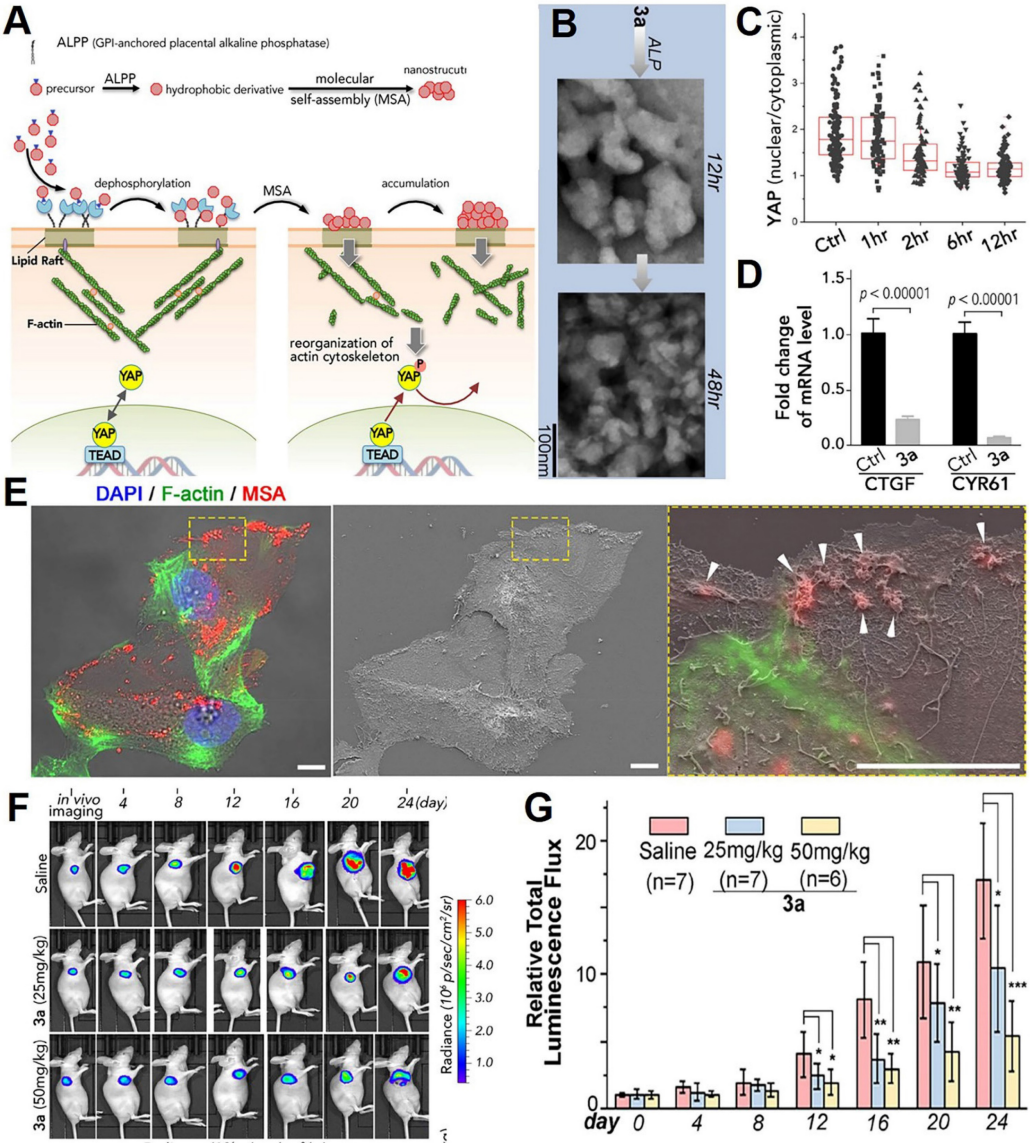
Design nanotherapeutics for interfering with the biomechanical transduction function of YAP. (A) Schematic illustrating the mechanisms of lipid-raft-targeted nanoplatforms for disturbing the YAP through actin cytoskeleton disruption. (B) Alkaline phosphatase dephosphorylation of 3a initiates molecular self-assembly at varying time points, forming diverse nanostructures. (C) Quantification of YAP intensity ratio between the nucleus and cytoplasm in SKOV3 cells at 60-70% confluence after incubation with 3a over different time periods. (D) The qPCR analysis of YAP target genes CTGF and CYR61 in untreated and 3a-treated SKOV3 cells. (E) Correlative light-electron microscopy of HeLa cells following incubation with 3a. Tumor growth was monitored (F) and analyzed (G) using bioluminescence detection. (Adapted with permission from Ref. 193. Copyright 2021 American Chemical Society)

The oncogenic activity of YAP is controlled by the Hippo kinase cascade and mechanical-force-induced actin remodeling. Li *et al*. from Okinawa Institute of Science and Technology Graduate University developed molecular self-assembly technology to selectively inhibit cancer cell proliferation by inactivating YAP (**Figure 7A**) 193. In this study, a ruthenium-complex-peptide precursor molecule was engineered to self-assemble into nanostructures under alkaline phosphatase action (**Figure 7B**). These nanostructures were designed to stabilize the lipid rafts of ovarian cancer cells. Upon stabilization, they trigger actin cytoskeleton remodeling (**Figure 7C**), with a particular focus on disrupting F-actin. This actin reorganization subsequently activates LATS, promoting YAP phosphorylation through Hippo signaling. To confirm YAP inactivation, time-lapse immunofluorescence staining of YAP in SKOV3 cells was conducted following 3a incubation, revealing clear inhibition of YAP nuclear translocation after 12 h (**Figure 7D**). Enhanced YAP phosphorylation deactivates YAP, suppressing TEAD-mediated target genes such as connective tissue growth factor (CTGF) and CYR61 (**Figure 7E**), ultimately inhibiting cancer cell proliferation *in vitro* and reducing ovarian tumor growth* in vivo*.

Molecular self-assembly technology has demonstrated strong anti-proliferative effects in various cancer cell lines and mouse xenograft models. In SKOV3-Luc xenograft mice, untreated tumors continued to grow throughout the 24-day observation period, while 3a-treated mice showed dose-dependent tumor suppression as early as 4 days post-injection. A 25 mg/kg dose of 3a reduced mean tumor volume by about 60% by day 16 compared to controls. By day 24, tumor volume was reduced by 45% and 60% in groups receiving 25 mg/kg and 50 mg/kg doses, respectively (**Figure 7F-G**). In summary, this strategy, which inhibits tumor growth by modulating YAP activity, offers a promising biomechanical regulatory approach to tumor nanotherapeutic strategy.

### Interfering cytoskeletal biomechanical sensing-transduction function

#### Electrostimulation disrupts cytoskeletal structure and function

**Figure 8:**
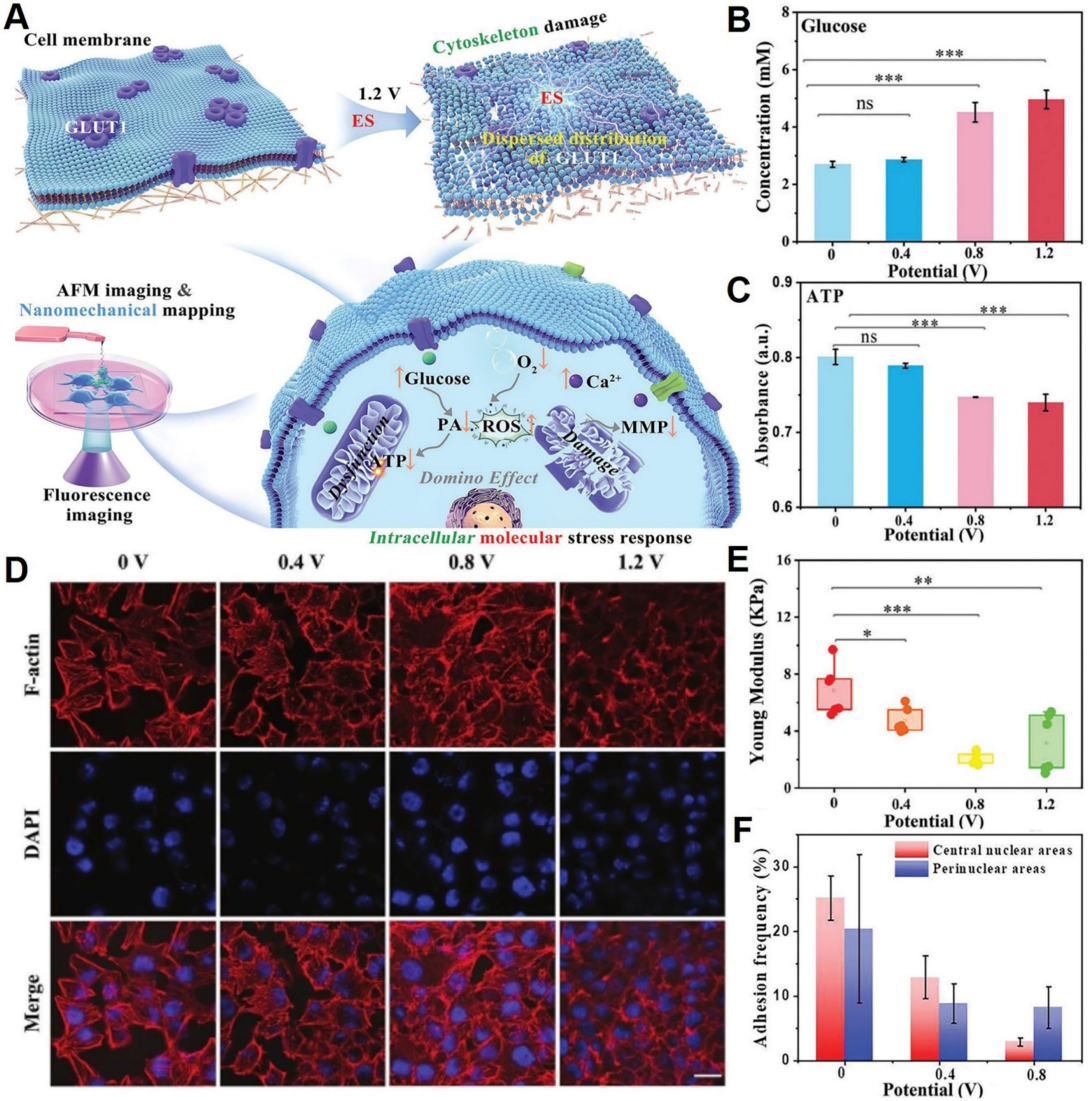
Electrostimulation disrupts the structure and function of the cytoskeleton. (A) Schematic representation of the molecular and nanomechanical insights into how ES inhibits energy metabolism and causes cytoskeletal damage in cancer cells. (B) Glucose concentration within MCF-7 cells measured using under different voltage conditions for 5 min. (C) ATP content in MCF-7 cells treated for 5 min at varying voltages. (D) Fluorescence imaging of MCF-7 cells subjected to different voltages for 5 min, showing F-actin (Cy3, red) and cell nuclei (DAPI, blue). (E) Statistical analysis of perinuclear Young's modulus (fitted using the Cone Sphere model) from MCF-7 cells exposed to different voltages for 5 min. (F) Probability statistics of GLUT1 recognition in the nuclear and perinuclear regions of MCF-7 cells after ES treatment at different voltages for 5 min. (Adapted with permission from Ref. 198. Copyright 2023 Wiley-VCH)

Based on the literature, it has been observed that tumor cells exhibit a comparatively higher susceptibility to external stimulation than normal cells, particularly with regards to their cytoskeletal structure 197. For instance, Jin's group from State Key Laboratory of Electroanalytical Chemistry of Chinese Academy of Sciences 198 demonstrated that electrostimulation (ES) significantly inhibits glucose and energy metabolism in cancer cells, resulting in rapid cell death (**Figure 8A-C**). From a mechanical perspective, ES leads to cytoskeletal disruption (**Figure 8D**), which reduces the Young's modulus of MCF-7 cell membranes (**Figure 8E**) due to the depolymerization of F-actin and the down regulation and irregular distribution of glucose transporter 1 (GLUT1) (**Figure 8F**). This effect highlights the potential of ES as a highly effective approach for clinical cancer treatments. Experiments reveal that high frequencies and cyclic pressures are primarily responsible for the disruption of actin fibers. Particularly, higher frequency and negative pressures in the latter half of the cycle induce greater tensile strain and deformation, leading to the breakdown of F-actin fibers and increased fluidization.

#### Low-intensity ultrasound disrupts cytoskeletal structure and function

As for another external stimulation, low-intensity ultrasound (LIUS) is widely used in medicine due to its non-invasive nature, safety, and ability to precisely target and manipulate biological tissues. The ultrasonic cavitation effect of LIUS involves the dynamic expansion and collapse of submicron air pockets, also known as cavitation nuclei, within a fluid when the sound pressure surpasses a certain threshold 199. The impact of LIUS on the cytoskeleton is pronounced, especially in tumor cells. Recently, Song *et al.* 200 discovered that Piezo1 plays a role in the apoptosis of pancreatic cancer cells when subjected to ultrasound (US) combined with microbubbles (MBs). However, since MBs used in this study are micron-sized, their ideal application *in vivo* presents certain challenges. Following treatment with US and MBs, tumors displayed slower growth rates; however, the growth rate remained higher in the US + MBs + Lv-siPiezo1 group compared to the US + MBs + Lv-NC group. This research emphasized the potential of using ultrasound alongside microbubbles as a non-invasive approach for treating pancreatic ductal adenocarcinoma through mechanotransduction. Additionally, other studies have shown that this combination can effectively disrupt the cytoskeletal structure of tumor cells by generating intense mechanical forces 201, 202. However, the micrometer size of MBs may limit their *in vivo* application, and achieving Piezo1 overexpression *in vivo* is challenging due to its high molecular weight. Overcoming these challenges will be crucial for future clinical applications.

### Interfering ECM-cellular membrane biomechanical sensing-transduction function

#### Interfering the mechanical properties of ECM

The TME exhibits increased stiffness due to an abundance of ECM, which amplifies its intrinsic mechanical properties 203. These 'inside-out' tensile forces are primarily mediated through integrin-dependent cell adhesions involving FAK activation 204. Consequently, targeting FAK in tumor tissue can modulate the mechanical properties of tumor and stromal cells as well as the tumor ECM. CRISPR/Cas genome editing offers substantial potential for cancer treatment by enabling precise inactivation or repair of cancer-related genes. A study developed multiplexed nanoparticles designed to deliver siFAK to disrupt the ECM, Cas9 mRNA to express Cas protein, and targeted sgRNA to knockout specific cancer genes 205. FAK inhibition was shown to reduce tumor cell contractility and membrane tension, along with ECM stiffness, thereby enhancing CRISPR gene editing efficiency in tumor cells both *in vitro* and *in vivo* by promoting lipid nanoparticles (LNPs) endocytosis and tumor penetration. *In vivo* results further demonstrated that siFAK + CRISPR-LNPs decreased metastatic potential in an ovarian cancer mouse model, improved outcomes in a tumor xenograft mouse model, and extended survival in an aggressive MYC-driven liver cancer model, highlighting significant anti-tumor effects across different cancer types.

#### Disrupting cellular membrane integrity

**Figure 9:**
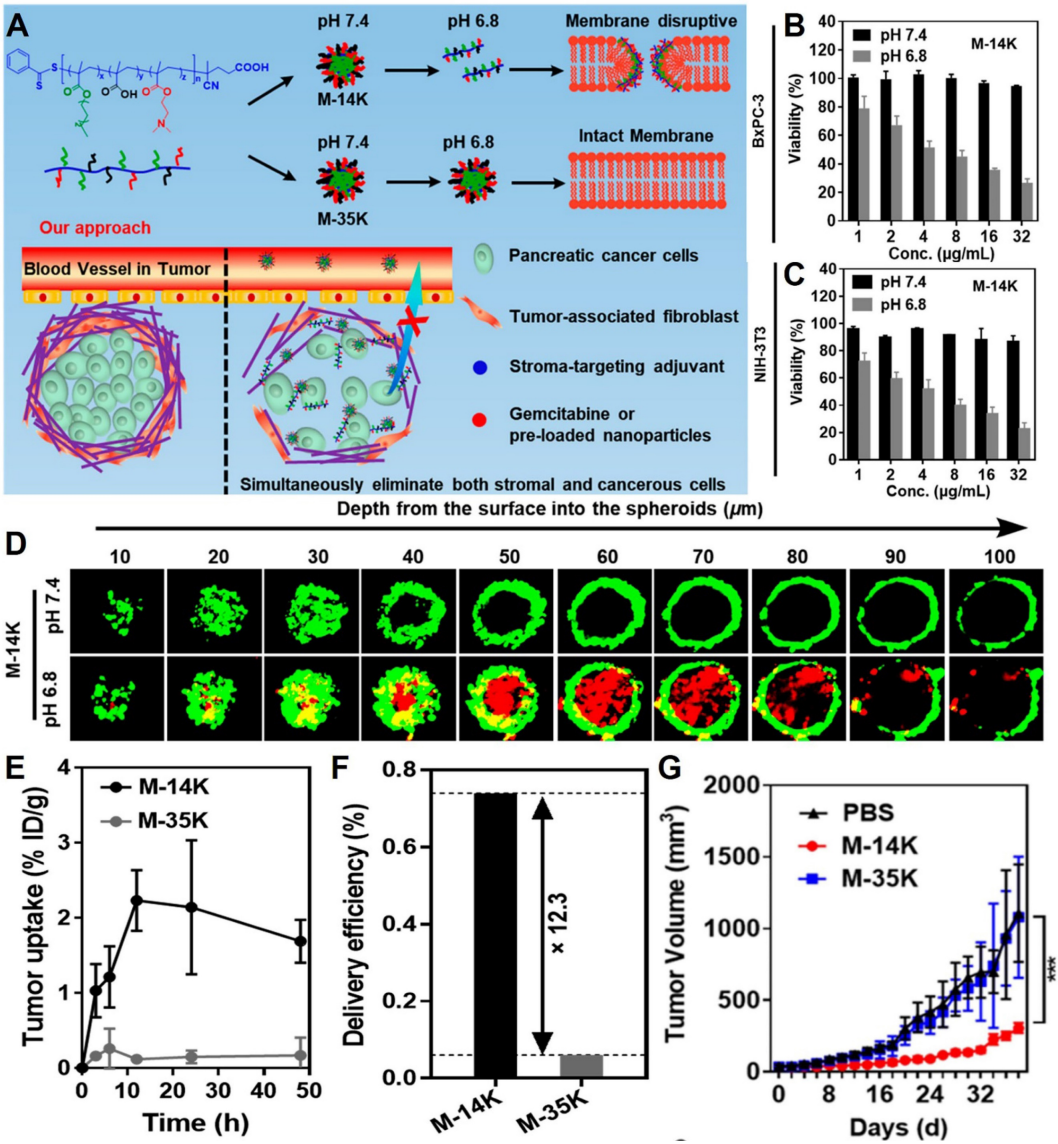
Design nanotherapeutics for disrupting the integrity of the cellular membrane. (A) Schematic of acid-activatable, membrane-disruptive nanomicelles (M-14K) designed to target both cancer and stromal cells. (B-C) Viability assays for BxPC-3 cancer cells and activated NIH-3T3 fibroblasts. (D) Images of three-dimensional BxPC-3@NIH-3T3 spheroids, showing a fibroblast shell (green) surrounding a core of cancer cells post-M-14K treatment; propidium iodide (red) stains the dead cells. (E) Tumor uptake of DiD-labeled M-14K and M-35K, and (F) comparison of calculated tumor-targeting efficiency between M-14K and M-35K in BxPC-3 tumor-bearing mouse models. (G) Tumor volume measurements during treatment. (Adapted with permission from Ref. 206. Copyright 2021 American Chemical Society)

The cellular membrane, composed of a lipid bilayer and cell surface receptors, detects mechanical signals from the environment and transmits this information to the intracellular cytoskeletal machinery. Thus, membrane-disruptive macromolecules can weaken membrane integrity, interfere with biomechanical signaling, and reduce the ability of cells to adhere to the stroma or neighboring cells. Yang's group from University of Science and Technology of China 206 demonstrated the feasibility of acid-responsive nanoparticles composed solely of membrane-disruptive molecules for treating pancreatic cancer with dense stromal barriers (**Figure 9A**). Using a pH-sensitive micelle derived from a polymeric mimic of host defense peptides as the core of the nanoplatform, the acid-activatable nanoparticle (M-14K) showed selective cytotoxicity toward BxPC-3 pancreatic cancer and NIH-3T3 fibroblast cells under mildly acidic conditions (**Figure 9B-C**). These nanoparticles dissociate at the weakly acidic pH of the TME (pH 6.5-6.8) but remain stable at physiological pH (7.4). In a BxPC-3@NIH-3T3 spheroid model, M-14K effectively penetrated the fibroblast layer to target cancer cells at pH 6.8 over 24 h (**Figure 9D**). Intravenous administration in mouse models with BxPC-3 xenograft tumors showed higher uptake of M-14K compared to its pH-insensitive counterpart (M-35K) (**Figure 9E**), with delivery efficiency 12.3 times that of M-35K (0.74% vs. 0.06%) (**Figure 9F**). Throughout the observation period, M-14K treatment significantly delayed tumor growth (**Figure 9G**) without causing off-target effects. Overall, this strategy provides a promising translational approach for improving pancreatic cancer treatment by disrupting cellular membrane integrity, permeating the stromal barrier, and interfering with biomechanical signaling. Although these pH-sensitive nanoplatforms show promise in treating pancreatic cancer, their non-biodegradability, limited cell selectivity, and model limitations remain significant drawbacks that must be addressed in future research.

### Interfering TME-biomechanical sensing-transduction function

**Figure 10:**
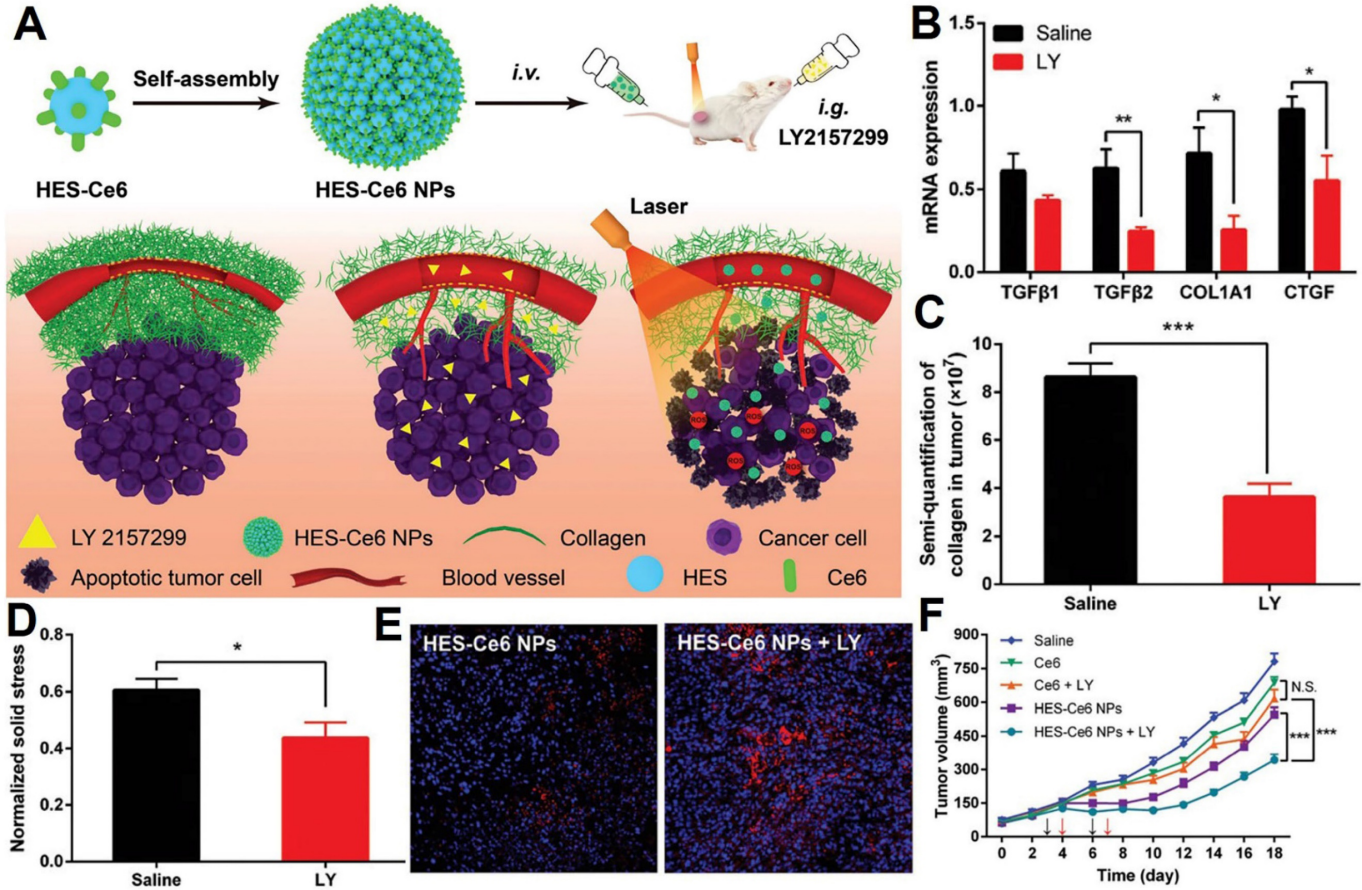
Design nanoplatforms for interfering with the biomechanical transduction function of the tumor microenvironment. (A) Schematic of HES-Ce6 NPs combined with TGFβ inhibitors to enhance PDT. (B) The mRNA expression levels of TGF-β1, TGF-β2, COL1A1, and CTGF in tumor tissues. (C) Semi-quantitative analysis of collagen in tumors using Masson staining. (D) Normalized solid stress measured as the ratio of tumor opening to tumor height. (E) Representative images of drug penetration in tumors (blue: DAPI, red: Ce6). (F) Tumor growth in 4T1 tumor-bearing mice. (Adapted with permission from Ref. 207. Copyright 2021 Royal Society of Chemistry)

In photodynamic therapy (PDT), the solid stress in stroma-rich tumors can hinder photosensitizer delivery. To address this, Chen *et al*. from Huazhong University of Science and Technology 207 proposed a strategy to enhance PDT efficacy by combining hydroxyethyl starch-chlorin e6 conjugate nanoparticles (HES-Ce6 NPs) with the TGF-β inhibitor LY2157299 (LY) (**Figure 10A**). Prior to PDT, LY administered intragastrically downregulated TGF-β signaling and ECM-related mRNA expression (**Figure 10B**), reduced collagen deposition (**Figure 10C**), alleviated solid stress (**Figure 10D**), and decompressed tumor blood vessels. This pretreatment significantly promoted HES-Ce6 NP penetration in tumors (**Figure 10E**), allowing the restructured tumor microenvironment to improve the accumulation and penetration of HES-Ce6 NPs, ultimately enhancing the anti-tumor efficacy of PDT (**Figure 10F**).

In a separate study, Cong *et al.* from Yanshan University 208 developed a “nano-lymphatic” system (DOX/g-C_3_N_4_/luminol@cytomembrane, DCL@M) aimed at addressing the elevated tumor IFP resulting from lymphatic insufficiency. In this system, lactic acid serves as a sacrificial agent, while DCL@M facilitates photocatalytic water splitting to reduce the volume of interstitial fluid, thereby mitigating the resistance to transfer caused by high tumor interstitial fluid pressure. The *in vivo* experiments demonstrated a significant 62.11% reduction in tumor IFP within the tumor tissue, which subsequently improved blood perfusion. The accumulation of the “nano-lymphatic” system (16.73%) in the tumor was found to be 15.9 times and 3.31 times greater than that of free doxorubicin hydrochloride (DOX, 1.05%) and DOX/g- C_3_N_4_@cytomembrane (DC@M, 3.03%), respectively. This indicates that the “nano-lymphatic” approach offers a novel strategy for enabling nanodrugs to navigate biological barriers and enhance therapeutic efficacy. Overall, these innovative strategies hold promise for advancing cancer treatments by overcoming the physical and mechanical challenges present within the tumor microenvironment. Although this research has highlighted the significant potential of the 'nano-lymphatic' system in tumor treatment, its clinical translation remains challenging. Issues such as biological safety, large-scale production difficulties, and individual patient variability can impact the efficacy of the 'nano-lymphatic' system, necessitating the integration of personalized treatment plans and precision medicine.

## Conclusions, challenges and prospectives

Tumors and tumor microenvironments complement each other, jointly promoting the growth, invasion, metastasis, and drug resistance of tumor cells. Therefore, effective tumor treatment strategies should regulate the tumor microenvironment simultaneously. Compared with chemical drugs, macromolecular drugs, and cell therapy, the main advantage of biomechanical based cancer treatment strategies is that: i) biomechanics can macroscopically regulate the function of cell secondary structures, such as primary cilia, cytoskeleton, etc., rather than targeting a single target or a specific type of cell. Therefore, the scope of regulation based on biomechanics is broad and has multiple impacts on tumor progression. For example, the regulation of the cytoskeleton can simultaneously affect DNA damage repair, metastasis, and drug resistance in tumor cells. This widespread effect makes the tumor suppressive effect stronger and less likely to develop tolerance. ii) The biomechanical regulation methods have the characteristic of diversity, which can be small molecule drugs or mechanical effects applied* in vitro*, such as low-intensity focused ultrasound, ultrasound cavitation, etc. Regulating tumor cells through physical means rather than chemically active biomolecules can significantly reduce common toxic side effects in tumor treatment, such as nausea, immune system suppression, and organ damage. However, the investigation of how biomechanics affect the onset and progression of cancer remains relatively underexplored. Mechanical imbalance is a significant feature of malignant tumor tissues, suggesting that disruptions in mechanical homeostasis may precede tumorigenesis and tumor advancement. A deeper exploration of biomechanics could facilitate earlier and more precise detection of cancer development and tumor formation, while also expanding the conversation about the various factors that contribute to cancer progression. This review presents a thorough overview of the known mechanical properties linked to malignant tumors. By synthesizing the molecular and mechanical characteristics at both cellular and tissue levels across different cancers, researchers can better focus on applying mechanobiology to the study of malignant conditions.

This paper analyzed the impact of the tumor mechanical environment on the occurrence and development of tumor angiogenesis, tumor drug resistance, and tumor metastasis. Mechanoreceptors initially detect mechanical signals from the TME and subsequently interact with mechanosignaling proteins to transduce these mechanical signals into biological signals, thereby modulating cellular responses, gene expression, and tumor microenvironment. The multidimensional mechanical forces experienced by tumors create abnormal tumor vasculature and morphological structures, leading to specific therapies such as nanodrug-mediated embolization treatment and tumor vasculature normalization induction strategies. All proteins acting as mechanosensors and the involved signaling networks have provided new therapeutic targets and challenges in overcoming tumor metastasis and drug resistance mechanisms.

Innovative bioengineering technologies and novel therapeutic strategies for biomechanical regulation offer transformative potential in tumor therapy by addressing the mechanical properties of cancer cells and their microenvironment. These technologies, such as targeted drug delivery systems and molecular self-assembly, enable precise control over cellular biomechanics, crucial for effective treatment. For instance, primary cilia-targeted nanoparticles enhance drug delivery and therapeutic efficacy by specifically targeting and modulating cilia functions. Additionally, advancements in mechanotransduction, such as using small molecules to inhibit key signaling pathways or employing low-intensity ultrasound to disrupt cytoskeletal structures, demonstrate significant promise in altering tumor progression and enhancing treatment outcomes. The integration of nanotechnology into biomechanical regulation strategies holds immense potential for revolutionizing cancer treatment. Ultimately, expanding research into mechanical properties and their impact on tumor behavior will enhance our understanding of cancer and lead to more effective, personalized therapies.

Nevertheless, the main challenge of biomechanical tumor treatment strategies lies in the translation of mechanobiological principles into clinical practice. Firstly, current research lacks simplifying and standardizing methods for measuring mechanical properties. The complexity of current technologies requires advancements to make them more accessible for clinical use. Secondly, while analyzing the adaptation of tumors to the surrounding mechanical environment, we also realize the significant gaps remain in our understanding of the complex interactions between mechanoreceptors, mechanosensors, and tumor progression. Current experimental models often fail to capture the dynamic mechanical interactions within tumors, highlighting the need for more sophisticated models. Thirdly, current research focuses on the therapeutic effect on tumors, while ignoring the safety of strategies based on mechanical signal interference. Subsequent research must further enhance the tumor targeting of therapeutic agents to reduce crosstalk with biomechanical signals of normal tissues. Finally, the therapeutic effects of combined treatment approaches based on tumor biomechanical regulation remain to be developed. For instance, prior to chemotherapy, physical methods like low-intensity ultrasound or electrical stimulation can be employed to disrupt the cytoskeletal structure of tumor cells, thereby reducing their drug resistance and enhancing the permeability and efficacy of chemotherapeutic agents. Similarly, inhibiting integrin-mediated cell-matrix adhesion signaling can decrease the adhesion force between tumor cells and the ECM, diminishing their ability to colonize other tissues. With the increasing attention and the continuous breakthrough of technical barriers, more patients will benefit from biomechanical regulation tumor therapeutic strategies.

## Figures and Tables

**Table 1:** Clinically used drug on mechanical forces of tumor treatment.

Drugs	Signaling pathway	Functional mechanism	Ref
PEGPH20	HA-tumor solid stress	Degrade HA and decrease solid stresses, enhance perfusion and drug delivery in pancreatic ductile adenocarcinomas	210
4-MU	HA-tumor solid stress	Inhibits HA synthesis by down-regulating HA receptors and the phosphatidylinositol 3-kinase/CD44 complex	88
A6	A6-CD44-HA	CD44 is a receptor for HA while A6 binds to CD44, resulting in the inhibition of the modulation of CD44-mediated cell signaling including HA	89
Bevacizumab	VEGFA-tumor angiogenesis	Prevents VEGFA from binding to receptors, hinders neovascularization and the activation of signal transduction cascades	32
Anlotinib	Tyrosine kinase inhibitors	Inhibit VEGFR, fbroblast growth factor receptors, platelet-derived growth factor receptors, c-Kit and Ret, resulting in inhibiting tumor angiogenesis and growth	211
α-solanine	EMT and MMPs	Blocking EMT and MMPs expression	212
Microtubule-destabilizing agents	Microtubule-cytoskeleton-mechanical forces	Inhibit microtubule polymerization at high concentrations, modulation of microtubule dynamics influence cytoskeleton	213
Microtubule-stabilizing agents	Microtubule	Promote microtubule polymerization	213
Mycalolide B	Actin-cytoskeleton	Inhibiting G-actin polymerization and severing F-actin by binding to barbed end of actin leads to a rapid collapse of the actin cytoskeleton, impairing cancer cell motility and invasion by blocking invadopodia-mediated ECM degradation	214
Collagenase	Collagen protein-stiffness of ECM	Decrease collagen proteins, reduce the stiffness of ECM, increase IgG diffusion to tumor sites in penetration-resistant tumors	68
GsMTx4	Piezo1	Inhibit the Ca^2+^ concentration, and alter EMT-correlated markers expression in response to mechanical stretch, influence the morphology and migration	215
shPTK2/PND1186	FAK	Represses YAP activation by inhibiting p-YAP^Y357^, leading to decreased YAP nuclear localization and activation, suppresses tumor initiation and progression	216
AZA1	Cdc42/RAC1 GTPase	Blocking Rac1/Cdc42-dependent cell cycle progression, cancer cell migration, and increase of cancer cell apoptosis involving down-regulation of the AKT and PAK signaling pathway	184
Pirfenidone	Antifibrotic	Restore biomechanical abnormalities of the tumor microenvironment, related to increased stiffness and hypo-perfusion	217
Tranilast	Antifibrotic	Reduce stiffness and mechanical forces, improve tumor perfusion and significantly enhance the efficacy of chemotherapy and nanomedicine by affecting CAFs	218
Ketotifen	Antifibrotic	Suppressed CAFs proliferation and stiffness of the extracellular matrix accompanied by an increase in vessel perfusion in fibrosarcoma and osteosarcoma	219
Losartan	Angiotensin inhibition	Reduces solid stress in tumours, resulting in increased vascular perfusion. And improves drug and oxygen delivery to tumours, thereby potentiating chemotherapy and reducing hypoxia in breast and pancreatic cancer models	35

**Table 2:** The classification and mechanisms of mechanobiology perturbing tumor nanotherapeutics.

Classification	Therapeutic mechanisms	Functional nanoplatform	Cargoes	Cancer type	Ref.
Interfering tumor microenvironment	Improve tumor blood vessel perfusion functionality	PEGylated liposomal	Tranilast and Doxil	Breast cancer	220
	Decrease collagen deposition, alleviated solid stress	Hydroxyethyl starch- Ce6 conjugate self-assembled nanoparticles	Ce6 and LY2157299	Breast cancer	207
	Decrease the volume of the tumor interstitial fluid to ameliorate the transfer resistance derived from the high tumor interstitial fluid pressure	Graphitic carbon nitride nanosheets	DOX and luminol	Cervical carcinoma	208
	Reduce mechanical stresses to decompress tumor vessels and improve perfusion and chemotherapy	Pegylated liposomal	DOX	Breast cancer	221
Interfering cell membrane	Decrease membrane tension and increase LNP endocytosis and tumor penetration.	Lipid nanoparticles	siRNA, mRNA, and targeted sgRNA	Ovarian cancer, and lung adenocarcinoma	205
	Acid-activatable disrupt cellular membrane integrity	Host defense peptides polymeric mimetic micelle	/	Pancreatic cancer	206
Interfering cytoskeletal	Reduce cell stiffness and inhibit cell migration through the graphene oxide nanosheets‑mediated disruption of the intracellular cytoskeleton	Graphene oxide nanosheets	/	Breast cancer	222
	Softening cells enhances nanoparticle uptake through activating clathrin- and caveolae-mediated endocytosis	Nitrogen-doped graphene quantum dots	DOX	Breast cancer	223
Interfering glycocalyx	Dynamic stretch forces combined with stiffness changes in the interstitium alter glycocalyx gene expression, thus change the cell uptake efficiency	Liposomal nanoparticles	DiI or DiO	Lung adenocarcinoma	224
Interfering primary ciliary	Inhibit primary cilia related signal lysophosphatidic acid signaling	PEG-PLGA nanoparticles	Ki16425	Glioblastoma	195
	Control the movement and function of a cilium with an external magnetic field, and improved cardiac function	CT-Fe_2_O_3_-NPs	Fenoldopam	LLC-PK1	196
Interfering mechanotransduction proteins	Inactivate Yes-associated protein and regulate Hippo signaling pathway	Ruthenium-complex-peptide precursor molecule	/	Ovarian cancer	193

**Table 3:** Clinical trials based on mechanical forces for tumor treatment.

Drugs	Cancer type	Indication	Tips	Ref
IAG933	Mesothelioma	NF2/LATS1/LATS2 mutated tumors and tumors with functional YAP/TAZ fusions	NCT04857372, Phase I, Recruiting	62
VT3989	Mesothelioma	Metastatic solid tumors that are resistant or refractory to standard therapy or for which no effective standard therapy	NCT04665206, Phase I, Recruiting	62
ION537	Advanced solid tumors	Molecularly selected advanced solid tumors	NCT04659096, Phase I, Completed	225
IK-930	Solid tumors	Malignant pleural mesothelioma, epithelioid hemangioendothelioma, NF2 deficient solid tumors, and solid tumors with YAP1/TAZ fusion genes	NCT05228015, Phase I, Terminated	226
VS-6063	Pancreatic Ductal adenocarcinoma	Resectable pancreatic ductal adenocarcinoma	NCT03727880, Phase II, Recruiting	227
VS-6766	Non-small cell lung cancer	Recurrent KRAS-mutant and BRAF-mutant non-small cell lung cancer	NCT04620330, Phase II, Completed	228
ADH-1	Melanoma	Advanced in-transit malignant melanoma	NCT00421811, Phase II, Completed	229
ADH-1	Solid tumors	Incurable solid tumors expressing N-cadherin	NCT00265057, Phase II, Completed	229
TG-0054	Hematological tumors	Multiple myeloma, and non-hodgkin lymphoma	NCT01458288, Phase II, Completed	230
PF-03732010	Solid tumors	Advanced solid tumors	NCT00557505, Phase I, Completed	231
CHM-2101	Advanced gastrointestinal cancer	Advanced gastrointestinal cancers resistant to at least one standard treatment in the metastatic or locally advanced setting.	NCT06055439, Phase I/II, Recruiting	232
Maraviroc	Colorectal cancer	Advanced colorectal cancer patients with hepatic liver metastases	NCT01736813	169
MBQ-167	Breast cancer	Breast cancer stage IV	NCT06075810, Phase I, Recruiting	233
SST0001	Multiple myeloma	Advanced refractory multiple myeloma	NCT01764880, Phase I, Completed	234

## Abbreviations

ECM: extracellular matrix
MS: mechanosensitive
AFM: atomic force microscopy
MPA: micropipette aspiration
TFM: traction force microscopy
TME: tumor microenvironment
MMPs: matrix metalloproteinases
HA: hyaluronic acid
IFP: interstitial fluid pressure
CAFs: cancer-associated fibroblasts
TAMs: tumor-associated macrophages
ECs: endothelial cells
HCC: hepatocellular carcinoma
PDAC: pancreatic ductal adenocarcinoma
MVP: microvascular pressure
GPCRs: G protein-coupled receptors
CTCs: circulating tumor cells
MTs: microtubules
ABC: ATP-binding cassette
YAP: yes-associated protein
TAZ: transcriptional coactivator with PDZ-binding motif
GCX: glycocalyx
GAG: glycosaminoglycan
HS: heparan sulfate
PC: primary cilia
PDAC: pancreatic ductal adenocarcinoma
PitNETs: pituitary neuroendocrine tumors
RAR: retinoic acid receptor
CREB: cAMP-response element binding protein
ATP: adenosine triphosphate
TRP: transient receptor potential
TRPM7: transient receptor potential melastatin 7
FAK: focal adhesion kinase
EMT: epithelial-mesenchymal transition
EGFR: epidermal growth factor receptor
CXCR4: C-X-C chemokine receptor type 4
CCR5: C-C chemokine receptor 5
GDP: guanosine diphosphate
GTP: guanosine triphosphate
ROCK: rho-associated coiled-coil-containing protein kinase
PAKs: p21-activated kinases
EPR: enhanced permeability and retention
LPAR1: lysophosphatidic acid receptor 1
LPA: lysophosphatidic acid
CT: cilia-targeted
GCX: glycocalyx
GAG: glycosaminoglycan
CTGF: connective tissue growth factor
ES: electrostimulation
GLUT1: glucose transporter 1
LIUS: low-intensity ultrasound
US: ultrasound
MBs: microbubbles
LNPs: lipid nanoparticles
M-14K: acid-activatable nanoparticle
PDT: photodynamic therapy
HES-Ce6 NPs: hydroxyethyl starch-chlorin e6 conjugate nanoparticles
LY: TGF-β inhibitor LY2157299
DCL@M: DOX/g-C_3_N_4_/luminol@cytomembrane
DOX: doxorubicin hydrochloride
DC@M: DOX/g- C_3_N_4_@cytomembrane
TGF-β: transforming growth factor-β
DP: guanosine diphosphate
GLUT1: glucose transporter 1
GO: graphene oxide

## References

[1] Pfister SX, Ashworth A (2017). Marked for death: targeting epigenetic changes in cancer. Nat Rev Drug Discov.

[2] Liu Q, Luo Q, Ju Y, Song G (2020). Role of the mechanical microenvironment in cancer development and progression. Cancer Biol Med.

[3] Händel C, Schmidt BUS, Schiller J, Dietrich U, Möhn T, Kießling TR (2015). Cell membrane softening in human breast and cervical cancer cells. New J Phys.

[4] Braig S, Sebastian Schmidt BU, Stoiber K, Händel C, Möhn T, Werz O (2015). Pharmacological targeting of membrane rigidity: implications on cancer cell migration and invasion. New J Phys.

[5] Massey A, Stewart J, Smith C, Parvini C, McCormick M, Do K (2024). Mechanical properties of human tumour tissues and their implications for cancer development. Nat Rev Phys.

[6] Suresh S (2007). Biomechanics and biophysics of cancer cells. Acta Biomater.

[7] Wu PH, Aroush DR, Asnacios A, Chen WC, Dokukin ME, Doss BL (2018). A comparison of methods to assess cell mechanical properties. Nat Methods.

[8] Gonzalez-Bermudez B, Guinea GV, Plaza GR (2019). Advances in micropipette aspiration: applications in cell biomechanics, models, and extended studies. Biophys J.

[9] Oh MJ, Kuhr F, Byfield F, Levitan I (2012). Micropipette aspiration of substrate-attached cells to estimate cell stiffness. J Vis Exp.

[10] Bustamante CJ, Chemla YR, Liu S, Wang MD (2021). Optical tweezers in single-molecule biophysics. Nat Rev Methods Primers.

[11] Youk JH, Gweon HM, Son EJ (2017). Shear-wave elastography in breast ultrasonography: the state of the art. Ultrasonography.

[12] Mariappan YK, Glaser KJ, Ehman RL (2010). Magnetic resonance elastography: a review. Clin Anat.

[13] Wang J, Lu D, Mao D, Long M (2014). Mechanomics: an emerging field between biology and biomechanics. Protein Cell.

[14] Kim YS, Majid M, Melchiorri AJ, Mikos AG (2019). Applications of decellularized extracellular matrix in bone and cartilage tissue engineering. Bioeng Transl Med.

[15] Bao L, Kong H, Ja Y, Wang C, Qin L, Sun H (2023). The relationship between cancer and biomechanics. Front Oncol.

[16] Stylianopoulos T, Martin JD, Chauhan VP, Jain SR, Diop-Frimpong B, Bardeesy N (2012). Causes, consequences, and remedies for growth-induced solid stress in murine and human tumors. Proc Natl Acad Sci U S A.

[17] Irvine KD, Shraiman BI (2017). Mechanical control of growth: ideas, facts and challenges. Development.

[18] Levayer R (2020). Solid stress, competition for space and cancer: The opposing roles of mechanical cell competition in tumour initiation and growth. Semin Cancer Biol.

[19] Tan M, Song B, Zhao X, Du J (2024). The role and mechanism of compressive stress in tumor. Front Oncol.

[20] Kim BG, Gao MQ, Kang S, Choi YP, Lee JH, Kim JE (2017). Mechanical compression induces VEGFA overexpression in breast cancer via DNMT3A-dependent miR-9 downregulation. Cell Death Dis.

[21] Jain RK, Martin JD, Stylianopoulos T (2014). The role of mechanical forces in tumor growth and therapy. Annu Rev Biomed Eng.

[22] Polacheck WJ, German AE, Mammoto A, Ingber DE, Kamm RD (2014). Mechanotransduction of fluid stresses governs 3D cell migration. Proc Natl Acad Sci U S A.

[23] Shields JD, Fleury ME, Yong C, Tomei AA, Randolph GJ, Swartz MA (2007). Autologous chemotaxis as a mechanism of tumor cell homing to lymphatics via interstitial flow and autocrine CCR7 signaling. Cancer Cell.

[24] Hyler AR, Baudoin NC, Brown MS, Stremler MA, Cimini D, Davalos RV (2018). Fluid shear stress impacts ovarian cancer cell viability, subcellular organization, and promotes genomic instability. PLoS One.

[25] Bates ME, Libring S, Reinhart-King CA (2023). Forces exerted and transduced by cancer-associated fibroblasts during cancer progression. Biol Cell.

[26] Pang MF, Siedlik MJ, Han S, Stallings-Mann M, Radisky DC, Nelson CM (2016). Tissue stiffness and hypoxia modulate the integrin-linked kinase ILK to control breast cancer stem-like cells. Cancer Res.

[27] Ghosh K, Thodeti CK, Dudley AC, Mammoto A, Klagsbrun M, Ingber DE (2008). Tumor-derived endothelial cells exhibit aberrant Rho-mediated mechanosensing and abnormal angiogenesis in vitro. Proc Natl Acad Sci U S A.

[28] Bordeleau F, Mason BN, Lollis EM, Mazzola M, Zanotelli MR, Somasegar S (2017). Matrix stiffening promotes a tumor vasculature phenotype. Proc Natl Acad Sci U S A.

[29] Wendong Y, Jiali J, Qiaomei F, Yayun W, Xianze X, Zheng S (2024). Biomechanical forces and force-triggered drug delivery in tumor neovascularization. Biomed Pharmacother.

[30] Dong Y, Xie X, Wang Z, Hu C, Zheng Q, Wang Y (2014). Increasing matrix stiffness upregulates vascular endothelial growth factor expression in hepatocellular carcinoma cells mediated by integrin β1. Biochem Biophys Res Commun.

[31] Wang Y, Zhang X, Wang W, Xing X, Wu S, Dong Y (2020). Integrin αVβ5/Akt/Sp1 pathway participates in matrix stiffness-mediated effects on VEGFR2 upregulation in vascular endothelial cells. Am J Cancer Res.

[32] Amadio M, Govoni S, Pascale A (2016). Targeting VEGF in eye neovascularization: What's new?: A comprehensive review on current therapies and oligonucleotide-based interventions under development. Pharmacol Res.

[33] Nia HT, Munn LL, Jain RK (2020). Physical traits of cancer. Science.

[34] Samuel T, Rapic S, O'Brien C, Edson M, Zhong Y, DaCosta RS (2023). Quantitative intravital imaging for real-time monitoring of pancreatic tumor cell hypoxia and stroma in an orthotopic mouse model. Sci Adv.

[35] Chauhan VP, Martin JD, Liu H, Lacorre DA, Jain SR, Kozin SV (2013). Angiotensin inhibition enhances drug delivery and potentiates chemotherapy by decompressing tumour blood vessels. Nat Commun.

[36] Boucher Y, Leunig M, Jain RK (1996). Tumor angiogenesis and interstitial hypertension. Cancer Res.

[37] Padera TP, Stoll BR, Tooredman JB, Capen D, di Tomaso E, Jain RK (2004). Pathology: cancer cells compress intratumour vessels. Nature.

[38] Grivennikov SI, Greten FR, Karin M (2010). Immunity, inflammation, and cancer. Cell.

[39] Lietz M, Dreesmann L, Hoss M, Oberhoffner S, Schlosshauer B (2006). Neuro tissue engineering of glial nerve guides and the impact of different cell types. Biomaterials.

[40] Jain RK (2013). Normalizing tumor microenvironment to treat cancer: bench to bedside to biomarkers. J Clin Oncol.

[41] Sun C, Jain RK, Munn LL (2007). Non-uniform plasma leakage affects local hematocrit and blood flow: implications for inflammation and tumor perfusion. Ann Biomed Eng.

[42] Pries AR, Höpfner M, le Noble F, Dewhirst MW, Secomb TW (2010). The shunt problem: control of functional shunting in normal and tumour vasculature. Nat Rev Cancer.

[43] Kamoun WS, Chae SS, Lacorre DA, Tyrrell JA, Mitre M, Gillissen MA (2010). Simultaneous measurement of RBC velocity, flux, hematocrit and shear rate in vascular networks. Nat Methods.

[44] Yuan F, Salehi HA, Boucher Y, Vasthare US, Tuma RF, Jain RK (1994). Vascular permeability and microcirculation of gliomas and mammary carcinomas transplanted in rat and mouse cranial windows. Cancer Res.

[45] Galie PA, Nguyen DH, Choi CK, Cohen DM, Janmey PA, Chen CS (2014). Fluid shear stress threshold regulates angiogenic sprouting. Proc Natl Acad Sci U S A.

[46] Coon BG, Baeyens N, Han J, Budatha M, Ross TD, Fang JS (2015). Intramembrane binding of VE-cadherin to VEGFR2 and VEGFR3 assembles the endothelial mechanosensory complex. J Cell Biol.

[47] Zanotelli MR, Reinhart-King CA (2018). Mechanical forces in tumor angiogenesis. In: Dong C, Zahir N, Konstantopoulos K, Eds. BIOMECHANICS IN ONCOLOGY, 1st ed. Cham: Springer.

[48] Yang S, Fei W, Zhao Y, Wang F, Ye Y, Wang F (2023). Combat against gynecological cancers with blood vessels as entry point: anti-angiogenic drugs, clinical trials and pre-clinical nano-delivery platforms. Int J Nanomedicine.

[49] Gensbittel V, Krater M, Harlepp S, Busnelli I, Guck J, Goetz JG (2021). Mechanical adaptability of tumor cells in metastasis. Dev Cell.

[50] Gargalionis AN, Papavassiliou KA, Papavassiliou AG (2022). Mechanobiology of solid tumors. Biochim Biophys Acta Mol Basis Dis.

[51] Zhovmer AS, Manning A, Smith C, Hayes JB, Burnette DT, Wang J (2021). Mechanical counterbalance of kinesin and dynein motors in a microtubular network regulates cell mechanics, 3D architecture, and mechanosensing. ACS Nano.

[52] Swaminathan V, Mythreye K, O'Brien ET, Berchuck A, Blobe GC, Superfine R (2011). Mechanical stiffness grades metastatic potential in patient tumor cells and in cancer cell lines. Cancer Res.

[53] Ferrara B, Pignatelli C, Cossutta M, Citro A, Courty J, Piemonti L (2021). The extracellular matrix in pancreatic cancer: description of a complex network and promising therapeutic options. Cancers (Basel).

[54] Yang Z, Zhou L, Si T, Chen S, Liu C, Ng KK (2023). Lysyl hydroxylase LH1 promotes confined migration and metastasis of cancer cells by stabilizing Septin2 to enhance actin network. Mol Cancer.

[55] Mistriotis P, Wisniewski EO, Bera K, Keys J, Li Y, Tuntithavornwat S (2019). Confinement hinders motility by inducing RhoA-mediated nuclear influx, volume expansion, and blebbing. J Cell Biol.

[56] Keys J, Cheung BCH, Elpers MA, Wu M, Lammerding J (2024). Rear cortex contraction aids in nuclear transit during confined migration by increasing pressure in the cell posterior. J Cell Sci.

[57] Ahn EH, Kim Y, Kshitiz, An SS, Afzal J, Lee S (2014). Spatial control of adult stem cell fate using nanotopographic cues. Biomaterials.

[58] Haschka MD, Karbon G, Soratroi C, O'Neill KL, Luo X, Villunger A (2020). MARCH5-dependent degradation of MCL1/NOXA complexes defines susceptibility to antimitotic drug treatment. Cell Death Differ.

[59] Diouf B, Crews KR, Lew G, Pei D, Cheng C, Bao J (2015). Association of an inherited genetic variant with vincristine-related peripheral neuropathy in children with acute lymphoblastic leukemia. Jama.

[60] van Tienderen GS, Rosmark O, Lieshout R, Willemse J, de Weijer F, Elowsson Rendin L (2023). Extracellular matrix drives tumor organoids toward desmoplastic matrix deposition and mesenchymal transition. Acta Biomater.

[61] Sleeboom JJF, van Tienderen GS, Schenke-Layland K, van der Laan LJW, Khalil AA, Verstegen MMA (2024). The extracellular matrix as hallmark of cancer and metastasis: From biomechanics to therapeutic targets. Sci Transl Med.

[62] Kalli M, Poskus MD, Stylianopoulos T, Zervantonakis IK (2023). Beyond matrix stiffness: targeting force-induced cancer drug resistance. Trends Cancer.

[63] Wu J, Chen J, Feng Y, Tian H, Chen X (2019). Tumor microenvironment as the "regulator" and "target" for gene therapy. J Gene Med.

[64] Barbazan J, Matic Vignjevic D (2019). Cancer associated fibroblasts: is the force the path to the dark side?. Curr Opin Cell Biol.

[65] Liu QP, Luo Q, Deng B, Ju Y, Song GB (2020). Stiffer matrix accelerates migration of hepatocellular carcinoma cells through enhanced aerobic glycolysis via the MAPK-YAP signaling. Cancers (Basel).

[66] Huang M, Wang H, Mackey C, Chung MC, Guan J, Zheng G (2023). YAP at the crossroads of biomechanics and drug resistance in human cancer. Int J Mol Sci.

[67] Medina SH, Bush B, Cam M, Sevcik E, DelRio FW, Nandy K (2019). Identification of a mechanogenetic link between substrate stiffness and chemotherapeutic response in breast cancer. Biomaterials.

[68] Netti PA, Berk DA, Swartz MA, Grodzinsky AJ, Jain RK (2000). Role of extracellular matrix assembly in interstitial transport in solid tumors. Cancer Res.

[69] Piccolo S, Panciera T, Contessotto P, Cordenonsi M (2023). YAP/TAZ as master regulators in cancer: modulation, function and therapeutic approaches. Nat Cancer.

[70] Najafi M, Farhood B, Mortezaee K (2019). Extracellular matrix (ECM) stiffness and degradation as cancer drivers. J Cell Biochem.

[71] Lee HO, Mullins SR, Franco-Barraza J, Valianou M, Cukierman E, Cheng JD (2011). FAP-overexpressing fibroblasts produce an extracellular matrix that enhances invasive velocity and directionality of pancreatic cancer cells. BMC Cancer.

[72] Kopecka J, Trouillas P, Gasparovic AC, Gazzano E, Assaraf YG, Riganti C (2020). Phospholipids and cholesterol: inducers of cancer multidrug resistance and therapeutic targets. Drug Resist Updat.

[73] Criscuolo D, Avolio R, Calice G, Laezza C, Paladino S, Navarra G (2020). Cholesterol homeostasis modulates platinum sensitivity in human ovarian cancer. Cells.

[74] Goebel A, Zinna VM, Dell'Endice S, Jaschke N, Kuhlmann JD, Wimberger P (2020). Anti-tumor effects of mevalonate pathway inhibition in ovarian cancer. BMC Cancer.

[75] Hendrich AB, Michalak K (2003). Lipids as a target for drugs modulating multidrug resistance of cancer cells. Curr Drug Targets.

[76] Preetha A, Banerjee R, Huilgol N (2007). Tensiometric profiles and their modulation by cholesterol: implications in cervical cancer. Cancer invest.

[77] Ye DM, Ye SC, Yu SQ, Shu FF, Xu SS, Chen QQ (2019). Drug-resistance reversal in colorectal cancer cells by destruction of flotillins, the key lipid rafts proteins. Neoplasma.

[78] Zalba S, Ten Hagen TL (2017). Cell membrane modulation as adjuvant in cancer therapy. Cancer Treat Rev.

[79] Chantemargue B, Di Meo F, Berka K, Picard N, Arnion H, Essig M (2018). Structural patterns of the human ABCC4/MRP4 exporter in lipid bilayers rationalize clinically observed polymorphisms. Pharmacol Res.

[80] Meyer dos Santos S, Weber CC, Franke C, Müller WE, Eckert GP (2007). Cholesterol: Coupling between membrane microenvironment and ABC transporter activity. Biochem Biophys Res Commun.

[81] Raghavan V, Vijayaraghavalu S, Peetla C, Yamada M, Morisada M, Labhasetwar V (2015). Sustained epigenetic drug delivery depletes cholesterol-sphingomyelin rafts from resistant breast cancer cells, influencing biophysical characteristics of membrane lipids. Langmuir.

[82] Tarbell JM, Cancel LM (2016). The glycocalyx and its significance in human medicine. J Intern Med.

[83] Sun Z, Guo SS, Fässler R (2016). Integrin-mediated mechanotransduction. J Cell Biol.

[84] Paszek MJ, Boettiger D, Weaver VM, Hammer DA (2009). Integrin clustering is driven by mechanical resistance from the glycocalyx and the substrate. PLoS Comput Biol.

[85] Paszek MJ, DuFort CC, Rossier O, Bainer R, Mouw JK, Godula K (2014). The cancer glycocalyx mechanically primes integrin-mediated growth and survival. Nature.

[86] Massey AE, Doxtater KA, Yallapu MM, Chauhan SC (2020). Biophysical changes caused by altered MUC13 expression in pancreatic cancer cells. Micron.

[87] Qazi H, Palomino R, Shi ZD, Munn LL, Tarbell JM (2013). Cancer cell glycocalyx mediates mechanotransduction and flow-regulated invasion. Integr Biol (Camb).

[88] Yates TJ, Lopez LE, Lokeshwar SD, Ortiz N, Kallifatidis G, Jordan A (2015). Dietary supplement 4-methylumbelliferone: an effective chemopreventive and therapeutic agent for prostate cancer. J Natl Cancer Inst.

[89] Finlayson M (2015). Modulation of CD44 Activity by A6-Peptide. Front Immunol.

[90] Zhao H, Wu L, Yan G, Chen Y, Zhou M, Wu Y (2021). Inflammation and tumor progression: signaling pathways and targeted intervention. Signal Transduct Target Ther.

[91] Olivo Pimentel V, Yaromina A, Marcus D, Dubois LJ, Lambin P (2020). A novel co-culture assay to assess anti-tumor CD8(+) T cell cytotoxicity via luminescence and multicolor flow cytometry. J Immunol Methods.

[92] (2021). Advancing cancer therapy. Nat Cancer.

[93] Mill P, Christensen ST, Pedersen LB (2023). Primary cilia as dynamic and diverse signalling hubs in development and disease. Nat Rev Genet.

[94] Khayyeri H, Barreto S, Lacroix D (2015). Primary cilia mechanics affects cell mechanosensation: A computational study. J Theor Biol.

[95] Higgins M, Obaidi I, McMorrow T (2019). Primary cilia and their role in cancer. Oncol Lett.

[96] Lee KH (2023). Primary cilia: a novel research approach to overcome anticancer drug resistance. Front Mol Biosci.

[97] Peixoto E, Jin S, Thelen K, Biswas A, Richard S, Morleo M (2020). HDAC6-dependent ciliophagy is involved in ciliary loss and cholangiocarcinoma growth in human cells and murine models. Am J Physiol Gastrointest Liver Physiol.

[98] Emoto K, Masugi Y, Yamazaki K, Effendi K, Tsujikawa H, Tanabe M (2014). Presence of primary cilia in cancer cells correlates with prognosis of pancreatic ductal adenocarcinoma. Hum Pathol.

[99] Martínez-Hernández R, Serrano-Somavilla A, Fernández-Contreras R, Sanchez-Guerrero C, Sánchez de la Blanca N, Sacristán-Gómez P (2024). Primary cilia as a tumor marker in pituitary neuroendocrine tumors. Mod Pathol.

[100] Kim SO, Kim BY, Lee KH (2022). Synergistic effect of anticancer drug resistance and Wnt3a on primary ciliogenesis in A549 cell-derived anticancer drug-resistant subcell lines. Biochem Biophys Res Commun.

[101] Sanyour HJ, Li N, Rickel AP, Torres HM, Anderson RH, Miles MR (2020). Statin-mediated cholesterol depletion exerts coordinated effects on the alterations in rat vascular smooth muscle cell biomechanics and migration. J Physiol.

[102] Krieg M, Dunn AR, Goodman MB (2014). Mechanical control of the sense of touch by β-spectrin. Nat Cell Biol.

[103] Pollard TD, Borisy GG (2003). Cellular motility driven by assembly and disassembly of actin filaments. Cell.

[104] Civelekoglu-Scholey G, Scholey JM (2010). Mitotic force generators and chromosome segregation. Cell Mol Life Sci.

[105] Tang Y, He Y, Zhang P, Wang J, Fan C, Yang L (2018). LncRNAs regulate the cytoskeleton and related Rho/ROCK signaling in cancer metastasis. Mol Cancer.

[106] Xin Y, Li K, Huang M, Liang C, Siemann D, Wu L (2023). Biophysics in tumor growth and progression: from single mechano-sensitive molecules to mechanomedicine. Oncogene.

[107] Chen X, Xu Z, Tang K, Hu G, Du P, Wang J (2023). The mechanics of tumor cells dictate malignancy via cytoskeleton-mediated APC/Wnt/β-catenin signaling. Research (Wash D C).

[108] Wirtz D, Konstantopoulos K, Searson PC (2011). The physics of cancer: the role of physical interactions and mechanical forces in metastasis. Nat Rev Cancer.

[109] Liu L, Jiang H, Zhao W, Meng Y, Li J, Huang T (2019). Cdc42-mediated supracellular cytoskeleton induced cancer cell migration under low shear stress. Biochem Biophys Res Commun.

[110] Tang K, Xin Y, Li K, Chen X, Tan Y (2021). Cell cytoskeleton and stiffness are mechanical indicators of organotropism in breast cancer. Biology-Basel.

[111] Heo SJ, Cosgrove BD, Dai EN, Mauck RL (2018). Mechano-adaptation of the stem cell nucleus. Nucleus.

[112] Kirby TJ, Lammerding J (2018). Emerging views of the nucleus as a cellular mechanosensor. Nat Cell Biol.

[113] Shi H, Zhou K, Wang M, Wang N, Song Y, Xiong W (2023). Integrating physicomechanical and biological strategies for BTE: biomaterials-induced osteogenic differentiation of MSCs. Theranostics.

[114] Swift J, Ivanovska IL, Buxboim A, Harada T, Dingal PC, Pinter J (2013). Nuclear lamin-A scales with tissue stiffness and enhances matrix-directed differentiation. Science.

[115] Malviya AN, Rogue PJ (1998). "Tell me where is calcium bred": clarifying the roles of nuclear calcium. Cell.

[116] Enyedi B, Jelcic M, Niethammer P (2016). The cell nucleus serves as a mechanotransducer of tissue damage-induced inflammation. Cell.

[117] Heo SJ, Thorpe SD, Driscoll TP, Duncan RL, Lee DA, Mauck RL (2015). Biophysical regulation of chromatin architecture instills a mechanical memory in mesenchymal stem cells. Sci Rep.

[118] Heo SJ, Han WM, Szczesny SE, Cosgrove BD, Elliott DM, Lee DA (2016). Mechanically induced chromatin condensation requires cellular contractility in mesenchymal stem cells. Biophys J.

[119] Greene JM, Schneble EJ, Jackson DO, Hale DF, Vreeland TJ, Flores M (2016). A phase I/IIa clinical trial in stage IV melanoma of an autologous tumor-dendritic cell fusion (dendritoma) vaccine with low dose interleukin-2. Cancer Immunol Immunother.

[120] Jain N, Iyer KV, Kumar A, Shivashankar GV (2013). Cell geometric constraints induce modular gene-expression patterns via redistribution of HDAC3 regulated by actomyosin contractility. Proc Natl Acad Sci U S A.

[121] Baarlink C, Wang H, Grosse R (2013). Nuclear actin network assembly by formins regulates the SRF coactivator MAL. Science.

[122] Denais CM, Gilbert RM, Isermann P, McGregor AL, te Lindert M, Weigelin B (2016). Nuclear envelope rupture and repair during cancer cell migration. Science.

[123] Dahl KN, Scaffidi P, Islam MF, Yodh AG, Wilson KL, Misteli T (2006). Distinct structural and mechanical properties of the nuclear lamina in Hutchinson-Gilford progeria syndrome. Proc Natl Acad Sci U S A.

[124] Nava MM, Miroshnikova YA, Biggs LC, Whitefield DB, Metge F, Boucas J (2020). Heterochromatin-driven nuclear softening protects the genome against mechanical stress-induced damage. Cell.

[125] dos Santos A, Toseland CP (2021). Regulation of nuclear mechanics and the impact on DNA damage. Int J Mol Sci.

[126] Panciera T, Azzolin L, Cordenonsi M, Piccolo S (2017). Mechanobiology of YAP and TAZ in physiology and disease. Nat Rev Mol Cell Biol.

[127] Shapovalov G, Ritaine A, Skryma R, Prevarskaya N (2016). Role of TRP ion channels in cancer and tumorigenesis. Semin Immunopathol.

[128] Chachisvilis M, Zhang YL, Frangos JA (2006). G protein-coupled receptors sense fluid shear stress in endothelial cells. Proc Natl Acad Sci U S A.

[129] He L, Si G, Huang J, Samuel ADT, Perrimon N (2018). Mechanical regulation of stem-cell differentiation by the stretch-activated Piezo channel. Nature.

[130] Cooper J, Giancotti FG (2019). Integrin Signaling in Cancer: Mechanotransduction, stemness, epithelial plasticity, and therapeutic resistance. Cancer Cell.

[131] Sulzmaier FJ, Jean C, Schlaepfer DD (2014). FAK in cancer: mechanistic findings and clinical applications. Nat Rev Cancer.

[132] Seetharaman S, Etienne-Manneville S (2018). Integrin diversity brings specificity in mechanotransduction. Biol Cell.

[133] Shen B, Delaney MK, Du X (2012). Inside-out, outside-in, and inside-outside-in: G protein signaling in integrin-mediated cell adhesion, spreading, and retraction. Curr Opin Cell Biol.

[134] Navab R, Strumpf D, To C, Pasko E, Kim KS, Park CJ (2016). Integrin α11β1 regulates cancer stromal stiffness and promotes tumorigenicity and metastasis in non-small cell lung cancer. Oncogene.

[135] Luo CW, Wu CC, Ch'ang HJ (2014). Radiation sensitization of tumor cells induced by shear stress: the roles of integrins and FAK. Biochim Biophys Acta.

[136] Lu P, Weaver VM, Werb Z (2012). The extracellular matrix: a dynamic niche in cancer progression. J Cell Biol.

[137] Zhang W, Wang J, Liu C, Li Y, Sun C, Wu J (2023). Crosstalk and plasticity driving between cancer-associated fibroblasts and tumor microenvironment: significance of breast cancer metastasis. J Transl Med.

[138] Levental KR, Yu H, Kass L, Lakins JN, Egeblad M, Erler JT (2009). Matrix crosslinking forces tumor progression by enhancing integrin signaling. Cell.

[139] Giancotti FG, Ruoslahti E (1990). Elevated levels of the alpha 5 beta 1 fibronectin receptor suppress the transformed phenotype of Chinese hamster ovary cells. Cell.

[140] Iwanicki MP, Chen H-Y, Iavarone C, Zervantonakis IK, Muranen T, Novak M (2016). Mutant p53 regulates ovarian cancer transformed phenotypes through autocrine matrix deposition. Jci Insight.

[141] Guo W, Giancotti FG (2004). Integrin signalling during tumour progression. Nat Rev Mol Cell Biol.

[142] Slack RJ, Macdonald SJF, Roper JA, Jenkins RG, Hatley RJD (2022). Emerging therapeutic opportunities for integrin inhibitors. Nat Rev Drug Discov.

[143] Zhang Y, Du J, Liu X, Shang F, Deng Y, Ye J (2024). Multi-domain interaction mediated strength-building in human alpha-actinin dimers unveiled by direct single-molecule quantification. Nat Commun.

[144] Drees F, Pokutta S, Yamada S, Nelson WJ, Weis WI (2005). Alpha-catenin is a molecular switch that binds E-cadherin-beta-catenin and regulates actin-filament assembly. Cell.

[145] Spill F, Reynolds DS, Kamm RD, Zaman MH (2016). Impact of the physical microenvironment on tumor progression and metastasis. Curr Opin Biotechnol.

[146] Yonemura S, Wada Y, Watanabe T, Nagafuchi A, Shibata M (2010). alpha-Catenin as a tension transducer that induces adherens junction development. Nat Cell Biol.

[147] Shen KH, Liao AC, Hung JH, Lee WJ, Hu KC, Lin PT (2014). alpha-Solanine inhibits invasion of human prostate cancer cell by suppressing epithelial-mesenchymal transition and MMPs expression. Molecules.

[148] Lawler K, O'Sullivan G, Long A, Kenny D (2009). Shear stress induces internalization of E-cadherin and invasiveness in metastatic oesophageal cancer cells by a Src-dependent pathway. Cancer Sci.

[149] Reid SE, Kay EJ, Neilson LJ, Henze AT, Serneels J, McGhee EJ (2017). Tumor matrix stiffness promotes metastatic cancer cell interaction with the endothelium. EMBO J.

[150] Benham-Pyle BW, Pruitt BL, Nelson WJ (2015). Cell adhesion. Mechanical strain induces E-cadherin-dependent Yap1 and beta-catenin activation to drive cell cycle entry. Science.

[151] Zhang Q, Lin F, Huang JY, Xiong CY (2022). Mechanical transmission enables EMT cancer cells to drive epithelial cancer cell migration to guide tumor spheroid disaggregation. Sci China Life Sci.

[152] Petho Z, Najder K, Bulk E, Schwab A (2019). Mechanosensitive ion channels push cancer progression. Cell Calcium.

[153] Coste B, Mathur J, Schmidt M, Earley TJ, Ranade S, Petrus MJ (2010). Piezo1 and Piezo2 are essential components of distinct mechanically activated cation channels. Science.

[154] Luo M, Cai G, Ho KKY, Wen K, Tong Z, Deng L (2022). Compression enhances invasive phenotype and matrix degradation of breast Cancer cells via Piezo1 activation. BMC Mol Cell Biol.

[155] Lai A, Cox CD, Chandra Sekar N, Thurgood P, Jaworowski A, Peter K (2022). Mechanosensing by Piezo1 and its implications for physiology and various pathologies. Biol Rev Camb Philos Soc.

[156] Chen X, Wanggou S, Bodalia A, Zhu M, Dong W, Fan JJ (2018). A feedforward mechanism mediated by mechanosensitive ion channel PIEZO1 and tissue mechanics promotes glioma aggression. Neuron.

[157] Tijore A, Yao M, Wang YH, Hariharan A, Nematbakhsh Y, Lee Doss B (2021). Selective killing of transformed cells by mechanical stretch. Biomaterials.

[158] Li X, Cheng Y, Wang Z, Zhou J, Jia Y, He X (2020). Calcium and TRPV4 promote metastasis by regulating cytoskeleton through the RhoA/ROCK1 pathway in endometrial cancer. Cell Death Dis.

[159] Momin A, Bahrampour S, Min HK, Chen X, Wang X, Sun Y (2021). Channeling force in the brain: mechanosensitive ion channels choreograph mechanics and malignancies. Trends Pharmacol Sci.

[160] Liu L, Wu N, Wang Y, Zhang X, Xia B, Tang J (2019). TRPM7 promotes the epithelial-mesenchymal transition in ovarian cancer through the calcium-related PI3K / AKT oncogenic signaling. J Exp Clin Cancer Res.

[161] Krzak G, Willis CM, Smith JA, Pluchino S, Peruzzotti-Jametti L (2021). Succinate receptor 1: an emerging regulator of myeloid cell function in inflammation. Trends Immunol.

[162] Dela Paz NG, Melchior B, Frangos JA (2017). Shear stress induces Gα(q/11) activation independently of G protein-coupled receptor activation in endothelial cells. Am J Physiol Cell Physiol.

[163] Li X, Wang J (2020). Mechanical tumor microenvironment and transduction: cytoskeleton mediates cancer cell invasion and metastasis. Int J Biol Sci.

[164] Yang N, Chen T, Wang L, Liu R, Niu Y, Sun L (2020). CXCR4 mediates matrix stiffness-induced downregulation of UBTD1 driving hepatocellular carcinoma progression via YAP signaling pathway. Theranostics.

[165] Dorsam RT, Gutkind JS (2007). G-protein-coupled receptors and cancer. Nat Rev Cancer.

[166] Liu Y, An S, Ward R, Yang Y, Guo XX, Li W (2016). G protein-coupled receptors as promising cancer targets. Cancer Lett.

[167] Lappano R, Maggiolini M (2017). Pharmacotherapeutic targeting of G protein-coupled receptors in oncology: examples of approved therapies and emerging concepts. Drugs.

[168] van Deventer HW, O'Connor W Jr, Brickey WJ, Aris RM, Ting JP, Serody JS (2005). C-C chemokine receptor 5 on stromal cells promotes pulmonary metastasis. Cancer Res.

[169] Halama N, Zoernig I, Berthel A, Kahlert C, Klupp F, Suarez-Carmona M (2016). Tumoral immune cell exploitation in colorectal cancer metastases can be targeted effectively by anti-CCR5 therapy in cancer patients. Cancer Cell.

[170] Almeria CVP, Setiawan IM, Siderius M, Smit MJ (2021). G protein-coupled receptors as promising targets in cancer. Curr Opin Endocr Metab Res.

[171] Hong SP, Yang MJ, Cho H, Park I, Bae H, Choe K (2020). Distinct fibroblast subsets regulate lacteal integrity through YAP/TAZ-induced VEGF-C in intestinal villi. Nat Commun.

[172] Qiao Y, Chen J, Lim YB, Finch-Edmondson ML, Seshachalam VP, Qin L (2017). YAP regulates actin dynamics through ARHGAP29 and promotes metastasis. Cell Rep.

[173] Zanconato F, Cordenonsi M, Piccolo S (2016). YAP/TAZ at the roots of cancer. Cancer Cell.

[174] Panciera T, Citron A, Di Biagio D, Battilana G, Gandin A, Giulitti S (2020). Reprogramming normal cells into tumour precursors requires ECM stiffness and oncogene-mediated changes of cell mechanical properties. Nat Mater.

[175] Nagelkerke A, Bussink J, Rowan AE, Span PN (2015). The mechanical microenvironment in cancer: how physics affects tumours. Semin Cancer Biol.

[176] Martellucci S, Clementi L, Sabetta S, Mattei V, Botta L, Angelucci A (2020). Src family kinases as therapeutic targets in advanced solid tumors: what we have learned so far. Cancers (Basel).

[177] Crosas-Molist E, Samain R, Kohlhammer L, Orgaz JL, George SL, Maiques O (2022). Rho GTPase signaling in cancer progression and dissemination. Physiol Rev.

[178] Sahai E, Marshall CJ (2002). RHO-GTPases and cancer. Nat Rev Cancer.

[179] Hodge RG, Ridley AJ (2016). Regulating Rho GTPases and their regulators. Nat Rev Mol Cell Biol.

[180] Khosravi-Far R, Solski PA, Clark GJ, Kinch MS, Der CJ (1995). Activation of Rac1, RhoA, and mitogen-activated protein kinases is required for Ras transformation. Mol Cell Biol.

[181] Qiu RG, Abo A, McCormick F, Symons M (1997). Cdc42 regulates anchorage-independent growth and is necessary for Ras transformation. Mol Cell Biol.

[182] Dyberg C, Fransson S, Andonova T, Sveinbjörnsson B, Lännerholm-Palm J, Olsen TK (2017). Rho-associated kinase is a therapeutic target in neuroblastoma. Proc Natl Acad Sci U S A.

[183] Prudnikova TY, Rawat SJ, Chernoff J (2015). Molecular pathways: targeting the kinase effectors of RHO-family GTPases. Clin Cancer Res.

[184] Zins K, Lucas T, Reichl P, Abraham D, Aharinejad S (2013). A Rac1/Cdc42 GTPase-specific small molecule inhibitor suppresses growth of primary human prostate cancer xenografts and prolongs survival in mice. PLoS One.

[185] Meng F, Na I, Kurgan L, Uversky VN (2016). Compartmentalization and functionality of nuclear disorder: intrinsic disorder and protein-protein interactions in intra-nuclear compartments. Int J Mol Sci.

[186] Amin R, Shukla A, Zhu JJ, Kim S, Wang P, Tian SZ (2021). Nuclear pore protein NUP210 depletion suppresses metastasis through heterochromatin-mediated disruption of tumor cell mechanical response. Nat Commun.

[187] Sun H, Dong Z, Zhang Q, Liu B, Yan S, Wang Y (2022). Companion-probe & race platform for interrogating nuclear protein and migration of living cells. Biosens Bioelectron.

[188] Zwerger M, Ho CY, Lammerding J (2011). Nuclear mechanics in disease. Annu Rev Biomed Eng.

[189] Zwerger M, Ho CY, Lammerding J (2011). Nuclear mechanics in disease. Annu Rev Biomed Eng, Vol 13.

[190] Poh YC, Shevtsov SP, Chowdhury F, Wu DC, Na S, Dundr M (2012). Dynamic force-induced direct dissociation of protein complexes in a nuclear body in living cells. Nat Commun.

[191] Tamiello C, Kamps MA, van den Wijngaard A, Verstraeten VL, Baaijens FP, Broers JL (2013). Soft substrates normalize nuclear morphology and prevent nuclear rupture in fibroblasts from a laminopathy patient with compound heterozygous LMNA mutations. Nucleus.

[192] Kaminski A, Fedorchak GR, Lammerding J (2014). The cellular mastermind(?)-mechanotransduction and the nucleus. Prog Mol Biol Transl Sci.

[193] Li G, Hu X, Nie P, Mang D, Jiao S, Zhang S (2021). Lipid-raft-targeted molecular self-assembly inactivates YAP to treat ovarian cancer. Nano Letters.

[194] Pala R, Mohieldin AM, Shamloo K, Sherpa RT, Kathem SH, Zhou J (2019). Personalized nanotherapy by specifically targeting cell organelles to improve vascular hypertension. Nano Letters.

[195] Loskutov YV, Griffin CL, Marinak KM, Bobko A, Margaryan NV, Geldenhuys WJ (2018). LPA signaling is regulated through the primary cilium: a novel target in glioblastoma. Oncogene.

[196] Pala R, Mohieldin AM, Sherpa RT, Kathem SH, Shamloo K, Luan Z (2019). Ciliotherapy: remote control of primary cilia movement and function by magnetic nanoparticles. Acs Nano.

[197] Samandari M, Abrinia K, Mokhtari-Dizaji M, Tamayol A (2017). Ultrasound induced strain cytoskeleton rearrangement: An experimental and simulation study. J Biomech.

[198] Qi G, Zhang M, Tang J, Jin Y (2023). Molecular/nanomechanical insights into electrostimulation-inhibited energy metabolism mechanisms and cytoskeleton damage of cancer cells. Adv Sci (Weinh).

[199] Yao H, Zhang L, Yan S, He Y, Zhu H, Li Y (2022). Low-intensity pulsed ultrasound/nanomechanical force generators enhance osteogenesis of BMSCs through microfilaments and TRPM7. J Nanobiotechnology.

[200] Song Y, Chen J, Zhang C, Xin L, Li Q, Liu Y (2022). Mechanosensitive channel Piezo1 induces cell apoptosis in pancreatic cancer by ultrasound with microbubbles. iScience.

[201] Singh A, Tijore A, Margadant F, Simpson C, Chitkara D, Low BC (2021). Enhanced tumor cell killing by ultrasound after microtubule depolymerization. Bioeng Transl Med.

[202] Van Steenbergen V, Boesmans W, Li Z, de Coene Y, Vints K, Baatsen P (2019). Molecular understanding of label-free second harmonic imaging of microtubules. Nat Commun.

[203] Mohammadi H, Sahai E (2018). Mechanisms and impact of altered tumour mechanics. Nat Cell Biol.

[204] Seong J, Wang N, Wang Y (2013). Mechanotransduction at focal adhesions: from physiology to cancer development. J Cell Mol Med.

[205] Zhang D, Wang G, Yu X, Wei T, Farbiak L, Johnson LT (2022). Enhancing CRISPR/Cas gene editing through modulating cellular mechanical properties for cancer therapy. Nat Nanotechnol.

[206] Fan F, Jin L, Yang L (2021). PH-sensitive nanoparticles composed solely of membrane-disruptive macromolecules for treating pancreatic cancer. ACS Appl Mater Interfaces.

[207] Chen J, Li S, Liu X, Liu S, Xiao C, Zhang Z (2021). Transforming growth factor-β blockade modulates tumor mechanical microenvironments for enhanced antitumor efficacy of photodynamic therapy. Nanoscale.

[208] Cong C, Rao C, Ma Z, Yu M, He Y, He Y (2020). "Nano-lymphatic" photocatalytic water-splitting for relieving tumor interstitial fluid pressure and achieving hydrodynamic therapy. Mater Horiz.

[209] Cambria E, Coughlin MF, Floryan MA, Offeddu GS, Shelton SE, Kamm RD (2024). Linking cell mechanical memory and cancer metastasis. Nat Rev Cancer.

[210] Wong KM, Horton KJ, Coveler AL, Hingorani SR, Harris WP (2017). Targeting the tumor stroma: the biology and clinical development of pegylated recombinant human hyaluronidase (PEGPH20). Curr Oncol Rep.

[211] Syed YY (2018). Anlotinib: first global approval. Drugs.

[212] Shen KH, Liao AC, Hung JH, Lee WJ, Hu KC, Lin PT (2014). α-Solanine inhibits invasion of human prostate cancer cell by suppressing epithelial-mesenchymal transition and MMPs expression. Molecules.

[213] Gudimchuk NB, McIntosh JR (2021). Regulation of microtubule dynamics, mechanics and function through the growing tip. Nat Rev Mol Cell Biol.

[214] Pipaliya BV, Trofimova DN, Grange RL, Aeluri M, Deng X, Shah K (2021). Truncated actin-targeting macrolide derivative blocks cancer cell motility and invasion of extracellular matrix. J Am Chem Soc.

[215] He J, Shan S, Li Q, Fang B, Xie Y (2022). Mechanical Stretch triggers epithelial-mesenchymal transition in keratinocytes through Piezo1 channel. Front Physiol.

[216] Song X, Xu H, Wang P, Wang J, Affo S, Wang H (2021). Focal adhesion kinase (FAK) promotes cholangiocarcinoma development and progression via YAP activation. J Hepatol.

[217] Mpekris F, Papaphilippou PC, Panagi M, Voutouri C, Michael C, Charalambous A (2023). Pirfenidone-loaded polymeric micelles as an effective mechanotherapeutic to potentiate immunotherapy in mouse tumor models. Acs Nano.

[218] Panagi M, Mpekris F, Chen P, Voutouri C, Nakagawa Y, Martin JD (2022). Polymeric micelles effectively reprogram the tumor microenvironment to potentiate nano-immunotherapy in mouse breast cancer models. Nat Commun.

[219] Panagi M, Mpekris F, Voutouri C, Hadjigeorgiou AG, Symeonidou C, Porfyriou E (2024). Stabilizing tumor-resident mast cells restores T-cell infiltration and sensitizes sarcomas to PD-L1 inhibition. Clin Cancer Res.

[220] Panagi M, Voutouri C, Mpekris F, Papageorgis P, Martin MR, Martin JD (2020). TGF-β inhibition combined with cytotoxic nanomedicine normalizes triple negative breast cancer microenvironment towards anti-tumor immunity. Theranostics.

[221] Papageorgis P, Polydorou C, Mpekris F, Voutouri C, Agathokleous E, Kapnissi-Christodoulou CP (2017). Tranilast-induced stress alleviation in solid tumors improves the efficacy of chemo- and nanotherapeutics in a size-independent manner. Sci Rep.

[222] Ghorbani M, Soleymani H, Hashemzadeh H, Mortezazadeh S, Sedghi M, Shojaeilangari S (2021). Microfluidic investigation of the effect of graphene oxide on mechanical properties of cell and actin cytoskeleton networks: experimental and theoretical approaches. Sci Rep.

[223] Chen X, Fan Y, Sun J, Zhang Z, Xin Y, Li K (2021). Nanoparticle-mediated specific elimination of soft cancer stem cells by targeting low cell stiffness. Acta Biomater.

[224] Kohon AI, Man K, Mathis K, Webb J, Yang Y, Meckes B (2023). Nanoparticle targeting of mechanically modulated glycocalyx. bioRxiv.

[225] Macleod AR (2021). Abstract ND11: The discovery and characterization of ION-537: A next generation antisense oligonucleotide inhibitor of YAP1 in preclinical cancer models. Cancer Res.

[226] Tolcher AW, Lakhani NJ, McKean M, Lingaraj T, Victor L, Sanchez-Martin M (2022). A phase 1, first-in-human study of IK-930, an oral TEAD inhibitor targeting the Hippo pathway in subjects with advanced solid tumors. J Clin Oncol.

[227] Abbas AA, Davelaar J, Gai J, Brown Z, Levi A, Linden S (2023). Preliminary translational immune and stromal correlates in a randomized phase II trial of pembrolizumab with or without defactinib for resectable pancreatic ductal adenocarcinoma (PDAC). J Clin Oncol.

[228] Capelletto E, Bironzo P, Denis L, Koustenis A, Bungaro M, Novello S (2022). Single agent VS-6766 or VS-6766 plus defactinib in KRAS-mutant non-small-cell lung cancer: the RAMP-202 phase II trial. Future Oncol.

[229] Neuendorf HM, Simmons JL, Boyle GM (2023). Therapeutic targeting of anoikis resistance in cutaneous melanoma metastasis. Front Cell Dev Biol.

[230] Yang J, Tian E, Chen L, Liu Z, Ren Y, Mao W (2024). Development and therapeutic perspectives of CXCR4 antagonists for disease therapy. Eur J Med Chem.

[231] Mescher M, Jeong P, Knapp SK, Rübsam M, Saynisch M, Kranen M (2017). The epidermal polarity protein Par3 is a non-cell autonomous suppressor of malignant melanoma. J Exp Med.

[232] Delaney S, Keinänen O, Lam D, Wolfe AL, Hamakubo T, Zeglis BM (2024). Cadherin-17 as a target for the immunoPET of adenocarcinoma. Eur J Nucl Med Mol Imaging.

[233] Medina JI, Cruz-Collazo A, Maldonado MD, Gascot TM, Borrero-Garcia LD, Cooke M (2022). Characterization of novel derivatives of MBQ-167, an inhibitor of the GTP-binding proteins Rac/Cdc42. Cancer Res Commun.

[234] Cassinelli G, Lanzi C, Tortoreto M, Cominetti D, Petrangolini G, Favini E (2013). Antitumor efficacy of the heparanase inhibitor SST0001 alone and in combination with antiangiogenic agents in the treatment of human pediatric sarcoma models. Biochem Pharmacol.

